# Spatial mapping of SARS-CoV-2 and H1N1 lung injury identifies differential transcriptional signatures

**DOI:** 10.1016/j.xcrm.2021.100242

**Published:** 2021-03-23

**Authors:** Camilla Margaroli, Paul Benson, Nirmal S. Sharma, Matthew C. Madison, Sarah W. Robison, Nitin Arora, Kathy Ton, Yan Liang, Liang Zhang, Rakesh P. Patel, Amit Gaggar

**Affiliations:** 1 Department of Medicine, Division of Pulmonary, Allergy & Critical Care Medicine, University of Alabama at Birmingham, Birmingham, AL, USA; 2 Program in Protease/Matrix Biology, University of Alabama at Birmingham, Birmingham, AL, USA; 3 Department of Pathology, Division of Anatomic Pathology, University of Alabama at Birmingham, Birmingham, AL, USA; 4 Department of Medicine, Division of Pulmonary, Allergy, and Critical Care Medicine, Brigham and Women’s Hospital, Boston, MA, USA; 5 Department of Pediatrics, Division of Neonatology University of Alabama at Birmingham and Children’s Hospital of Alabama, Birmingham, AL, USA; 6 Nanostring Technologies Inc., Seattle, WA, USA; 7 Department of Pathology, Division of Molecular & Cellular Pathology, University of Alabama at Birmingham, Birmingham, AL, USA; 8 Center for Free Radical Biology, University of Alabama at Birmingham, Birmingham, AL, USA; 9 Birmingham VA Medical Center, Birmingham, AL, USA

**Keywords:** SARS-CoV-2, COVID-19, H1N1 influenza, ARDS, spatial transcriptomics

## Abstract

Severe SARS-CoV-2 infection often leads to the development of acute respiratory distress syndrome (ARDS), with profound pulmonary patho-histological changes post-mortem. It is not clear whether ARDS from SARS-CoV-2 is similar to that observed in influenza H1N1, another common viral cause of lung injury. Here, we analyze specific ARDS regions of interest utilizing a spatial transcriptomic platform on autopsy-derived lung tissue from patients with SARS-CoV-2 (n = 3), H1N1 (n = 3), and a dual infected individual (n = 1). Enhanced gene signatures in alveolar epithelium, vascular tissue, and lung macrophages identify not only increased regional coagulopathy but also increased extracellular remodeling, alternative macrophage activation, and squamous metaplasia of type II pneumocytes in SARS-CoV-2. Both the H1N1 and dual-infected transcriptome demonstrated an enhanced antiviral response compared to SARS-CoV-2. Our results uncover regional transcriptional changes related to tissue damage/remodeling, altered cellular phenotype, and vascular injury active in SARS-CoV-2 and present therapeutic targets for COVID-19-related ARDS.

## Highlights

SARS-CoV-2 and H1N1-induced ARDS show unique transcriptional signatures
Tissue remodeling pathways dominate the transcriptional profile of SARS-CoV-2 ARDS
SARS-CoV-2/H1N1 double infection resembles the lung anti-viral response to H1N1

## Graphical abstract

**Figure:**
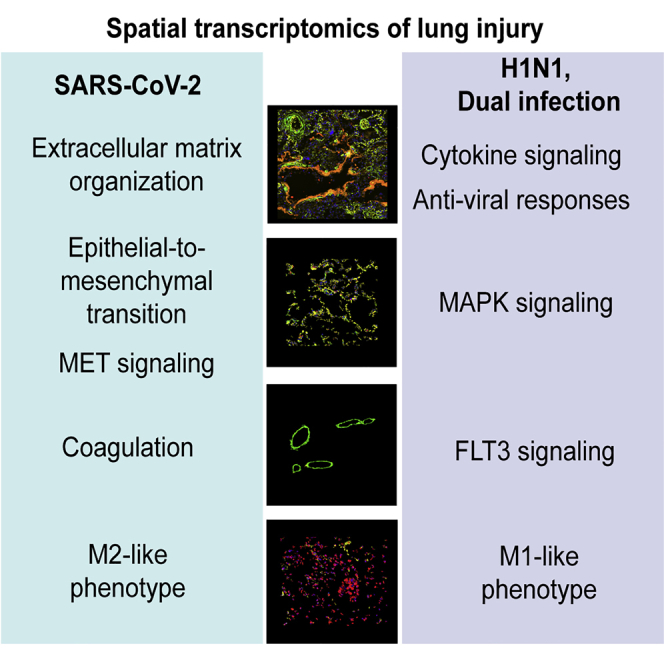

## Teaser

Lung injury can be induced by viral infections, such as H1N1 and SARS-CoV-2 infections. Here, Margaroli et al. investigate differential lung responses to both viruses. SARS-CoV-2-induced genes relate to tissue remodeling, while the H1N1 profile is skewed toward anti-viral responses.

## Introduction

Acute respiratory distress syndrome (ARDS) has been observed following respiratory viral infections, most notably during H1N1 influenza pandemics^1^ and during the current COVID-19 pandemic caused by the the severe acute respiratory syndrome-coronavirus 2 (SARS-CoV-2).^2^^,^^3^ Both SARS-CoV-2 and H1N1-mediated ARDS have been characterized by increased lung inflammation and increased disease-related morbidity and mortality.^4^, ^5^, ^6^ However, there has been recent evidence suggesting that SARS-CoV-2 patients have extended hospitalizations in subjects with ARDS compared to influenza-induced ARDS,^7^ indicating that the cellular processes that drive this pathology may differ between these two important viral causes of lung injury.

Post-mortem lung and vascular tissues from SARS-CoV-2 subjects have shown profound morphological changes,^8^^,^^9^ with diffuse alveolar damage, induction of fibrotic responses in the lung epithelium,^10^ and presence of vascular congestion and thrombi.^8^^,^^11^ Furthermore, temporal and spatial heterogeneity of lung responses to SARS-CoV-2 have been reported,^12^ including differential expression of interferon gamma in patients with high viral load. However, the transcriptional alterations identified while preserving the tissue architecture and within the lungs of SARS-CoV-2 ARDS subjects compared to other viral forms of ARDS remain poorly defined. There are also limited data related to potential co-infection of both viruses leading to ARDS,^13^ as there has been little influenza during the current fall/winter period. Here, we analyzed the histological and transcriptional response of key structural and immune cells, while preserving the tissue architecture of the lung, in ARDS patients infected with SARS-CoV-2, H1N1, and an individual who was infected with both viruses.

## Results

### COVID-19 ARDS lung has a discrete regional transcriptomic profile

To investigate similarities and regional differences in viral-induced ARDS, we used autopsy lung tissue from seven patients diagnosed with ARDS and confirmed SARS-CoV-2 or H1N1 infection (Table S1). We leveraged the GeoMX Digital Spatial Profiling platform to delineate and sequence-specific regions of interest (ROIs) from tissues, *in situ* hybridized with an 1860 gene platform (GeoMX COVID-19 Immune Response Atlas) (STAR Methods; Figure S1).

**Figure 1:**
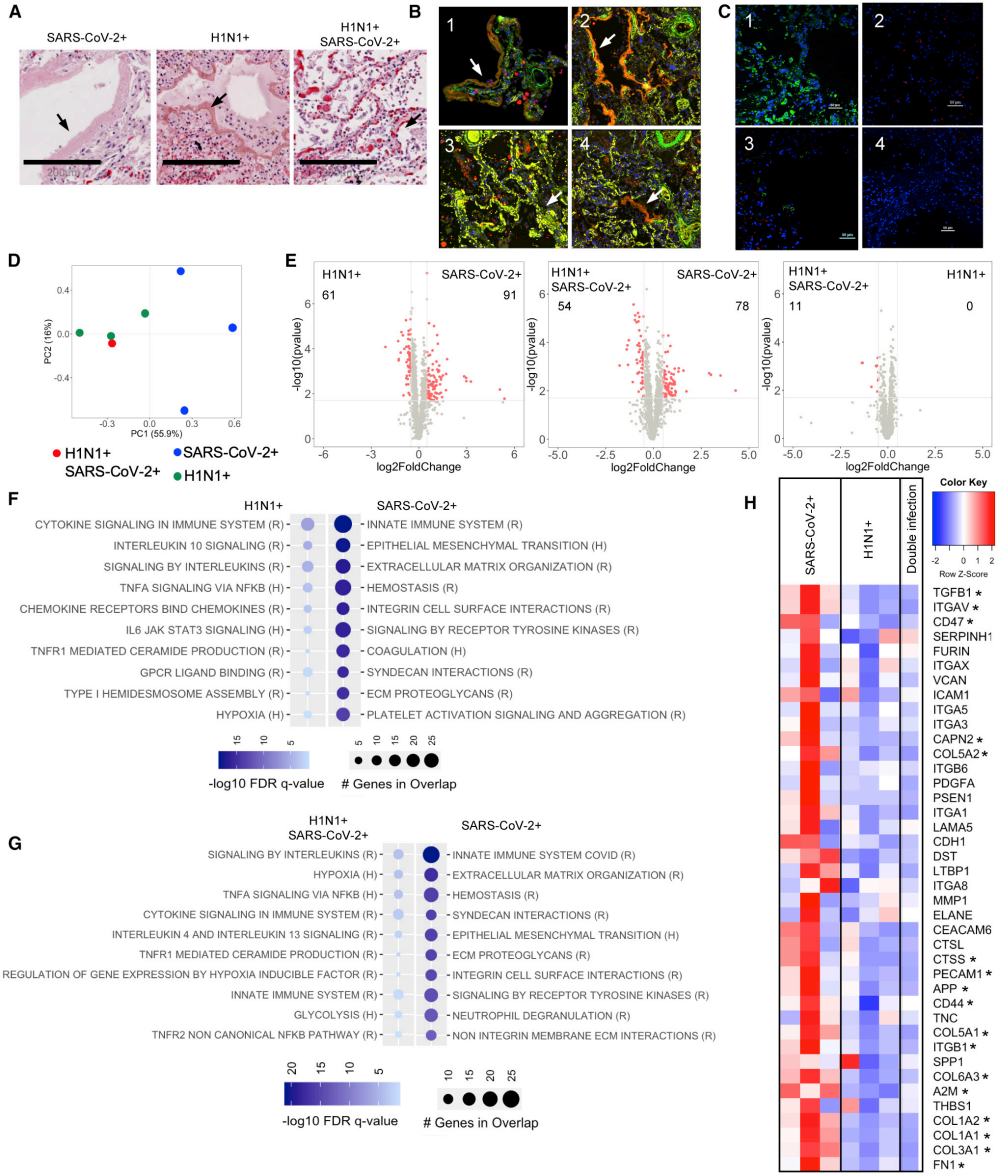
SARS-CoV-2-induced lung injury shows a discrete transcriptional signature (A) Histological analysis of tissues sections stained by H&E (scale bars, 200 μm) revealed presence of ARDS in all three patient groups; arrows indicate hyaline membranes. (B) Immunofluorescent staining (10× magnification) of α-SMA (green), CD68 (red), and EpCAM (yellow) in SARS-CoV-2 (1), H1N1 (2), double infected (3), and areas of low viral load (4); arrows indicate hyaline membranes. (C) Immunofluorescent staining of SARS-CoV-2 (green), H1N1 (red), and DAPI (blue) in SARS-CoV-2 (1), H1N1 (2), double infected (3), and areas of low viral load (4). Scale bars, 50 μm. (D) PCA analysis of transcriptional signatures in total lung injury. (E–G) Differential gene-expression analysis and gene set enrichment analysis (GSEA) using reactome (R) and hallmark (H) datasets for upregulated or downregulated genes in SARS-CoV-2-infected patients (n = 3) compared to (F) H1N1 (n = 3) or (G) SARS-CoV-2/H1N1 (n = 1). Differential gene expression was defined as p = 0.02 and log2 fold change of 0.5. (H) Heatmap representation of genes involved in tissue remodeling and their relative expression in all three types of infection (asterisk indicates significant genes between H1N1 and SARS-CoV-2 shown in the volcano plot).

All patients displayed histologic regions of ARDS defined as diffused alveolar damage (DAD) and by the presence of alveolar epithelial injury (hyaline membranes) (Figure 1A).^14^ These regions were subsequently stained by immunofluorescence to delineate epithelial (EpCAM^+^), vascular (smooth muscle actin^+^), and macrophage populations (CD68^+^) (Figure 1B) and selected for transcriptional profiling. Further, these regions were stained with antibodies directed to SARS-CoV-2 or influenza A to identify areas of increased versus low viral burden (Figure 1C).

The selected ARDS regions, which delineated the transcriptional response of the total lung tissue, demonstrated differences in transcriptome expression between the SARS-CoV-2 versus both H1N1 alone and dual viral infection regions (Figures 1D and 1E). Although immune activation pathways were found to predominate in the H1N1 (Figure 1F) and dual-infected regions (Figure 1G), SARS CoV-2 regions demonstrated increased expression of epithelial-to-mesenchymal transition (EMT), coagulation, and extracellular matrix (ECM) pathways. This notable increase in ECM-related genes in SARS-CoV-2 included increased ECM components, ECM-related signaling molecules, and proteases (Figure 1H), which resulted in presence of higher collagen deposition in the SARS-CoV-2 lung tissue compared to H1N1 (Figure S2). Interestingly, the gene expression of virus-rich versus virus-poor regions within each infection group did not show significant differences (Figure S3).

To further identify transcriptional signatures of structural lung cells and resident immune cells that may contribute to the ARDS phenotype, cells of the vascular bed (smooth muscle actin^+^), epithelium (EpCAM^+^), and macrophage populations (CD68^+^) were selected by immunofluorescent staining. The purity of the selected populations and their RNA was validated by the expression of cell-specific genes (Figure S4A): PDGFRB (vascular),^15^ KRT7 (epithelium),^16^ and CD68 (macrophages),^17^ which followed the expected trends. Furthermore, using immunofluorescence we assessed protein expression of one of the most upregulated genes in SARS-CoV-2 patients compared to H1N1 in each cell type (Figure S4B), showing co-localization of the protein with the selected cell type.

### COVID-19 ARDS-associated vascular beds show increased hypercoagulopathy

**Figure 2:**
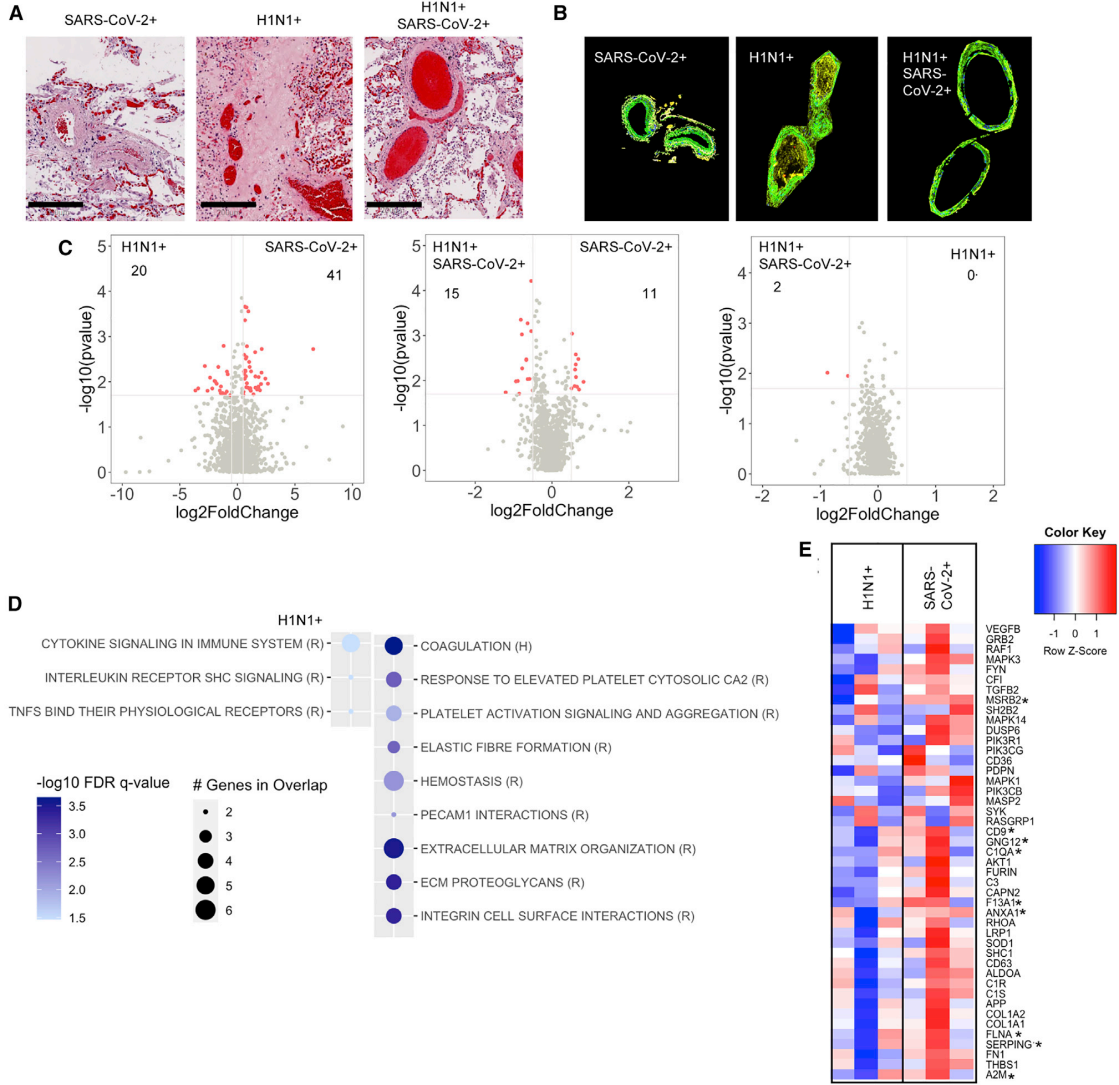
SARS-CoV-2 infection induces a hypercoagulopathy transcriptional program in the pulmonary vascular bed (A and B) Histological analysis of the lung vascular bed stained (A) by H&E (scale bars, 200 μm) or (B) by immunofluorescence for α-SMA (green). (C and D) Differential gene-expression analysis (C) and GSEA using reactome (R) and hallmark (H) datasets (D) for upregulated or downregulated genes in SARS-CoV-2-infected patients compared to H1N1. Differential gene expression was defined as p = 0.02 and log2 fold change of 0.5. (E) Heatmap representation of genes involved in coagulation, complement, and platelet activation and their relative expression in all SARS-CoV-2 and H1N1 infection (asterisk indicates significant genes between H1N1 and SARS-CoV-2 shown in the volcano plot). SARS-CoV-2-infected patients (n = 3), H1N1 (n = 3), and SARS-CoV-2/H1N1 (n = 1).

Due in part to recent evidence of hypercoagulopathy in SARS-CoV-2 subjects,^18^ we examined vascular beds proximal to regions of DAD (Figures 2A and 2B). Although H1N1 and dual-infected vascular regions demonstrated relatively similar gene expression (Figure 2C, right panel), there were notable differences when these groups were compared to SARS-CoV-2 regions (Figure 2C, left and middle panel). Pathway analyses demonstrated an increased expression of ECM and coagulation-related genes in SARS-CoV-2 versus increased immune signaling in H1N1 and dual-infected vascular cells (Figure 2D; Figure S5A). Genes related to complement activation, platelet biology, and endothelial injury were greatly enhanced in SARS-CoV-2 vascular beds (Figure 2E), demonstrating a transcriptionally regulated response promoting hypercoagulopathy.

### Alveolar epithelium in COVID-19 displays squamous metaplasia of type II pneumocytes

**Figure 3:**
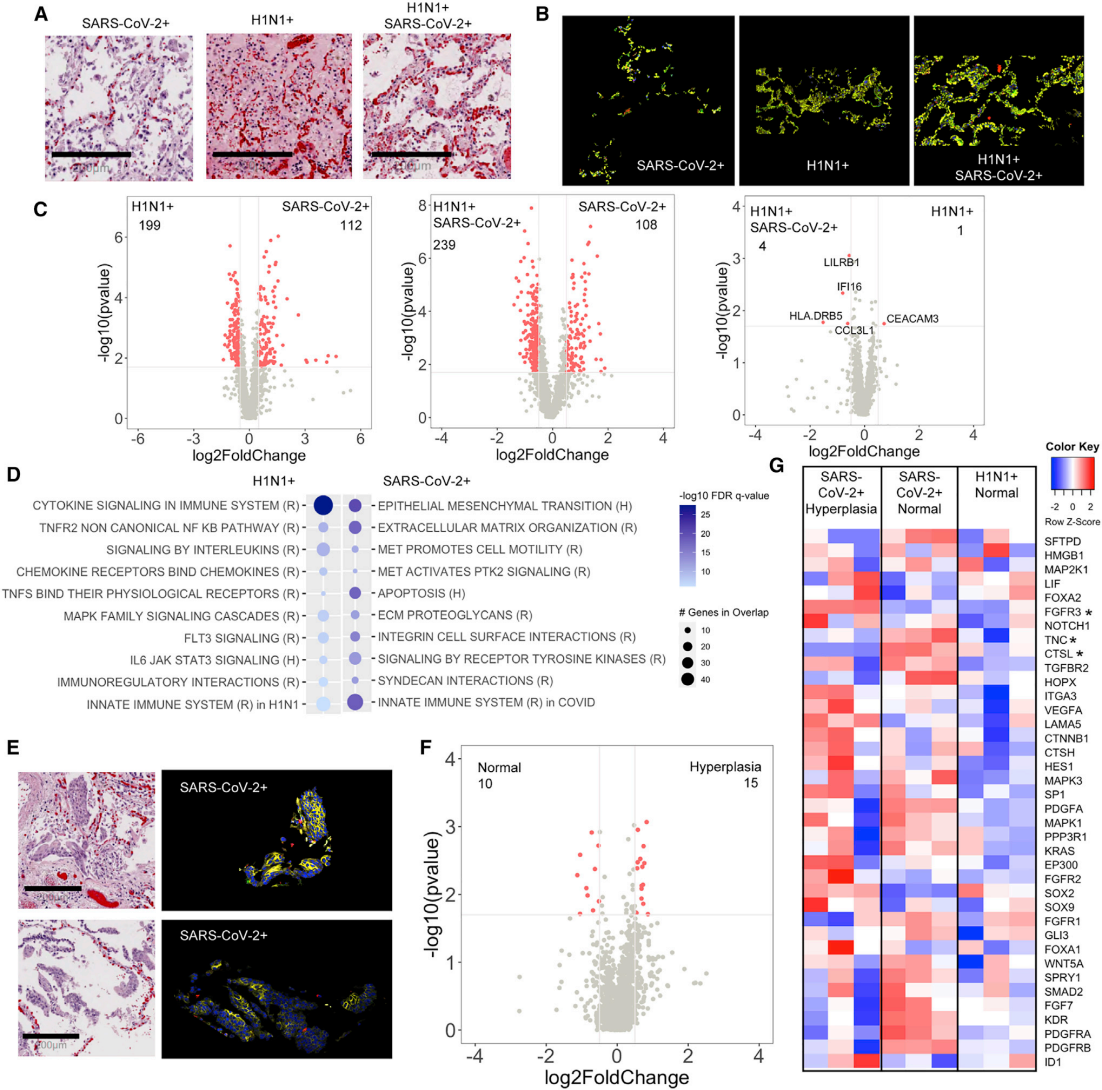
SARS-CoV-2 infection promotes alveolar epithelial hyperplasia (A and B) Histological analysis of the alveolar epithelium stained (A) by H&E (scale bars, 200 μm) or (B) by immunofluorescence for EpCAM (yellow). (C and D) Differential gene-expression analysis (C) and GSEA using reactome (R) and hallmark (H) datasets (D) for upregulated or downregulated genes in SARS-CoV-2-infected patients compared to H1N1. (E) Histological analysis of alveolar epithelium (scale bars, 200 μm for H&E) in SARS-CoV-2 patients shows cellular hyperplasia in H&E with EpCAM^+^ immunofluorescent staining (yellow). (F) Differential gene-expression analysis of normal and hyperplastic alveolar epithelium in SARS-CoV-2-infected patients. (G) Heatmap representation of genes involved in alveolar epithelium proliferation (GO:0060502) and their relative expression in all SARS-CoV-2 normal alveolar epithelium, hyperplastic alveolar epithelium, and H1N1 normal alveolar epithelium (asterisk indicates significant genes between H1N1 and SARS-CoV-2 shown in the volcano plot). Differential gene expression was defined as p = 0.02 and log2 fold change of 0.5. SARS-CoV-2-infected patients (n = 3), H1N1 (n = 3), and SARS-CoV-2/H1N1 (n = 1).

We next sought to characterize the alveolar epithelium, a site of notable damage in ARDS (Figures 3A and 3B).^19^ As observed with the vascular regions, there was little differential gene expression between dual-infected and H1N1-infected epithelial regions (Figure 3C, right panel). However, there were significant differences in gene expression with SARS-CoV-2 compared to the H1N1 and dual-infected epithelial regions (Figure 3C, left and middle panel). Gene-pathway analyses showed that, although immune signaling was observed in all groups, SARS-CoV-2 regions demonstrated increased EMT and ECM-related pathways (Figure 3D; Figure S5A). Further, we noted that SARS-CoV-2 subjects did demonstrate areas of increased alveolar epithelial hyperplasia (Figure 3E), a phenotype that has recently been described in COVID-19 autopsy specimens.^8^ When compared to non-hyperplastic epithelial regions, we observed no significant difference in gene expression (Figure 3F). However, when we compared specific genes related to lung epithelial proliferation and squamous metaplasia of type II pneumocytes in the SARS-CoV-2 hyperplastic and normal epithelium with the non-hyperplastic H1N1 epithelium, we observed increased expression of those genes in the SARS-CoV-2 regions (Figure 3G). These results suggest that cellular metaplasia is an important feature of SARS-CoV-2 ARDS.

### Macrophages are differentially activated in COVID-19

**Figure 4:**
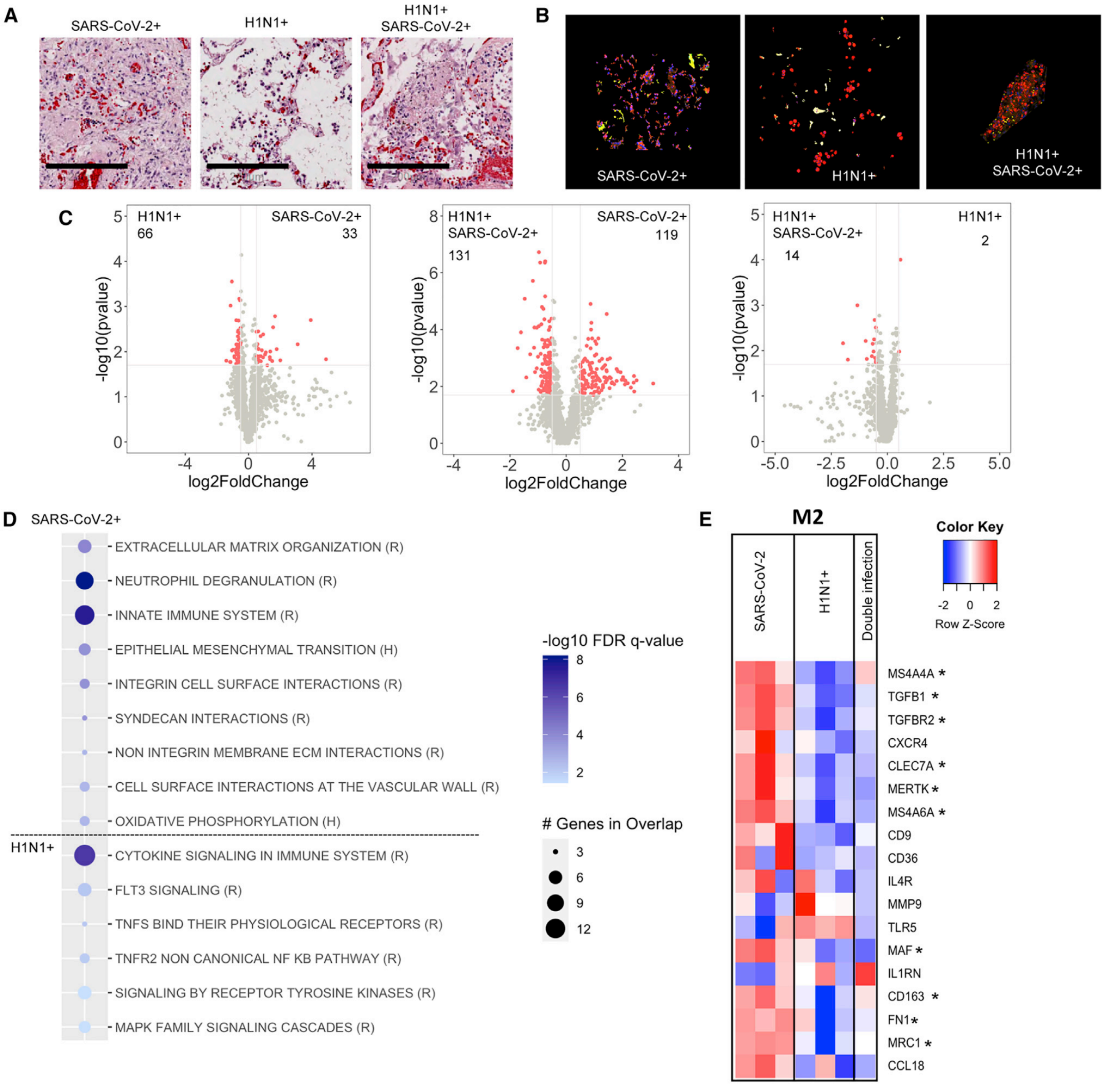
SARS-CoV-2 infection induces an alternative activation phenotype in lung macrophages (A and B) Histological analysis of the lung macrophages (scale bars, 200 μm) stained by H&E (A) and immunofluorescence for CD68 (red) (B). (C and D) Differential gene-expression analysis (C) and GSEA using reactome (R) and hallmark (H) datasets (D) for upregulated or downregulated genes in SARS-CoV-2-infected patients compared to H1N1. (E) Heatmap representation of genes defining a pro-inflammatory (M1) or alternative activated (M2) macrophage phenotype (asterisk indicates significant genes between H1N1 and SARS-CoV-2 shown in the volcano plots). Differential gene expression was defined as p = 0.02 and log2 fold change of 0.5. SARS-CoV-2-infected patients (n = 3), H1N1 (n = 3), and SARS-CoV-2/H1N1 (n = 1).

As macrophages are critical to the immune response to pulmonary viral infections,^20^ we next identified macrophages by H&E and CD68 staining (Figures 4A and 4B). We noted that SARS-CoV-2 patients displayed two phenotypes for pulmonary macrophages: either within clusters or infiltrative phenotype (Figures S6A and S6B). However, when these groups were compared with each other, there were only eight genes that were differentially regulated (Figure S6C) suggesting that these groups are of similar lineage.

There was little differential gene expression in macrophages between the dual infected and H1N1 infected (Figure 4C, right panel). However, there were significant differences in macrophage gene expression in SARS-CoV-2 compared to H1N1 regions and the dual infected compared to SARS-CoV-2 (Figure 4C, left and middle panels). Gene-pathway analyses showed that, although immune signaling was observed in all groups, SARS-CoV-2 regions demonstrated enrichment of pathways related to tissue remodeling and differential activation of the innate immune signaling (Figure 4D; Figure S5B). SARS-CoV-2 macrophages displayed genes consistent with alternative macrophage activation^21^ (Figure 4E).

## Discussion

Our study focused on examining transcriptional signatures active in viral ARDS from H1N1 and SARS-CoV-2 patients to better understand fundamental biologic processes related to these critical disease phenotypes. Although prior transcriptomic studies have focused on individual immune cells,^22^^,^^23^ isolated epithelial cells,^24^ or bulk lung tissue RNA,^25^ these approaches do not have the benefit of maintaining tissue architecture to examine gene expression *in situ*. To date, only the study from Desai et al.^12^ addresses the heterogeneous responses in the SARS-CoV-2 lung while maintaining an intact tissue architecture. In this study, we utilized the spatial transcriptomic approach to focus on ARDS regions in SARS-CoV-2 subjects compared to other forms of virus-induced ARDS, providing the ability to identify regional or cell-specific gene regulation.

Prior studies have largely delineated the expression of altered coagulopathy as a significant gene signature.^9^^,^^11^^,^^26^ Although our results validate these findings, using spatial transcriptomics, we also identify a notable enrichment in extracellular-matrix-associated genes in SARS-CoV-2 regions compared to H1N1. Prior data have shown that excessive ECM turnover portends increased mortality in other causes of ARDS.^27^ Interleukin-6 (IL-6) has been associated with worse outcomes in patients with SARS-CoV-2.^28^^,^^29^ In line with the study by Desai et al.,^12^ we did not find major differences within the SARS-CoV-2 cohort for IL-6 expression between patients and regions of differential viral presence. It has been previously shown that IL-6 increases transforming growth factor-β (TGF-β) signaling by modulating the compartmentalization of the TGF-β receptor,^30^ which is a known pro-fibrotic signaling pathway. Interestingly, in our dataset genes involved in the IL-6 signaling pathway were enriched in H1N1 patients. This differential expression may be due to diverse temporal regulation of tissue responses in these two viral infections, where early high IL-6 signaling in SARS-CoV-2 induces a quicker fibroproliferative cascade leading to more severe cases of lung injury and a higher collagen deposition observed in the terminal cases in this study.

Overall, these results challenge conventional wisdom and provide evidence for a more fibroproliferative ARDS phenotype in SARS-CoV-2 infection versus a more exudative inflammatory ARDS phenotype in H1N1 infection. This finding may explain the extended time on ventilator support often required by COVID-19 ARDS patients.^31^ As we are biased with only terminal ARDS subjects, it is possible that this gene signature may represent a unique endotype for COVID-19,^32^ which progresses to poorer outcomes versus other COVID-19 subjects who recover from ARDS. Our results also strongly suggest that, for these COVID-19 ARDS subjects, directed anti-fibrotic therapies may provide an important therapeutic approach to improve disease-related outcomes. Furthermore, this clear differential regulation of disease pathology warrants future studies in the temporal regulation of immune responses to airborne viruses.

The transcriptional signature from isolated cell populations were also consistent with these larger ARDS regions. ARDS-associated capillaries and arterioles from SARS-CoV-2-infected subjects show a notable upregulation of coagulopathy, complement activation, and platelet aggregation genes, highlighting that regional vessels are actively transcriptionally contributing to the development of COVID-associated vascular injury and microangiopathy.^33^ Alveolar epithelial cells from SARS-CoV-2 subjects demonstrated regions of increased hyperplasia, a finding observed in multiple patients in a recent autopsy study.^8^ It is interesting that both hyperplastic and non-hyperplastic SARS-CoV-2 alveolar epithelium had similar transcriptome, but both had enhanced metaplasia-related gene expression compared to H1N1 alveolar epithelia. A recent manuscript has suggested that EMT may be protective in early SARS-CoV-2 infection by reducing ACE2 expression;^34^ its role in later-stage ARDS may be to induce a wound-healing response that has become dysregulated.

Macrophages have previously been shown to be central to immune response with SARS-CoV-2, and previous data suggest that they are critical to a “cytokine storm” in early SARS-CoV-2 infection.^35^ Our data confirm that, in end-stage ARDS, both SARS-CoV-2 and H1N1 lungs demonstrated increased immune activation pathways, but the SARS-CoV-2 regions demonstrated alternative macrophage activation. These data, together with a lack of differences in gene expression observed between ARDS regions characterized with either increased or low viral staining, suggest that the patients included in this study may be part of the low-viral-load group described in the study by Desai et al.^12^ In that study, patients with higher viral loads presented a macrophage phenotype skewed toward an M1-like activation paired with increased interferon responses. Furthermore, they suggest that a broad, tissue-based transcriptome response in this patient group may not impacted by viral presence during end-stage ARDS in both SARS-CoV-2- and H1N1-infected patients.

Overall, these results provide further evidence of a more fibroproliferative response to the SARS-CoV-2 versus H1N1. Future studies should also work to define the transcriptome and activity of both innate^36^ and adaptive^37^^,^^38^ immunity in the lungs of patients with persistent viral infection and injury and their relative contributions to the SARS-CoV-2 ARDS phenotype. Furthermore, future studies should also address the mechanisms leading to the discrepant transcriptional response observed in H1N1 and SARS-CoV-2 ARDS, as the autopsy specimens used in this study limit the ability to address the kinetics of differential ARDS development.

We also identified a co-infected individual who, upon virus staining, demonstrated a much higher burden of H1N1 compared to SARS-CoV-2 throughout the lungs. This may underscore why this individual’s transcriptome more closely related to the H1N1 subjects. It is important to note that this subject had known lung disease with CT-ILD and COPD and was treated with baseline methotrexate and steroids prior to hospitalization, which may have made this individual more susceptible to dual viral infection. A small clinical series of co-infections with SARS-CoV-2 and influenza has been reported,^39^ and, in these three cases, there was no obvious evidence of baseline immunosuppression noted. Although the current influenza season has seen reduced cases of hospitalized patients, it is imperative to consider that if both viruses are highly prevalent in the future, co-infection in both immunosuppressed and immunocompetent individuals may be a more frequent occurrence.

This method of analysis has uncovered pathways that augment tissue injury in SARS-CoV-2 individuals, and future studies should examine additional critically ill subjects and focus on further defining these critical pathways. It is our hope that these pathways will improve our understanding of mechanisms leading to progressive worsening of gas exchange and increased mortality in SARS-CoV-2-related ARDS. As a result, the potential for new therapeutic targets to alter the fibroproliferative response will present the potential to improve clinical outcomes in patients with progressive lung injury.

### Limitations of study

The numbers of subjects included in this study are relatively limited, even though the SARS-CoV-2 and H1N1 subjects were relatively well matched for demographic features, severity of disease, and co-morbidities. These features strongly suggest that the transcriptional changes observed were due to the viral lung injury, although future studies with additional subjects would certainly be warranted. Furthermore, this study focused only on the lung parenchyma, and future studies addressing spatial heterogeneity (larger and smaller airways, lung parenchyma versus lumen) are needed. Finally, patient samples used in this study represent a discrete patient population, where disease was most severe. Future studies addressing the temporal regulation of disease will help better understand the differences observed in these two viral infections.

## STAR★Methods

### Key resources table

**Table:** 

REAGENT or RESOURCE	SOURCE	IDENTIFIER
**Antibodies**		

SARS-CoV-2 nucleocapsid	GeneTex	Cat#: GTX135361 RRID: AB_2887484
H1N1	Abcam	Cat#ab20343, RRID:AB_445525
CD68	Abcam	Cat# ab224029, clone:EPR20545
EpCAM	Abcam	Cat# ab213500, clone:EPR220532-222
alpha-SMA	Invitrogen	Cat# 53-9760-82, RRID:AB_2574461
KRT19	Thermofisher	Cat# TA803863, clone: OTI6A8
TGFBR2	Thermofisher	Cat# TA807912, clone: OTI3B4
F13A1	Abcam	Cat# ab225018, clone: EP3372
CD68	Santa Cruz	Cat# sc-20060 AF647, RRID:AB_627158

**Biological samples**		

Patient paraffine embedded lung tissue	UAB Tissue Biorepository Core Facility	https://sites.uab.edu/tissuebank/

**Chemicals, peptides, and recombinant proteins**		

Xylene	Fisher Scientific	Cat#X5P-1GAL
100% Ethanol	Fisher Scientific	Cat#HC-800-1GL
95% Ethanol	Fisher Scientific	Cat#HC-1100-1GL
Tris-EDTA ph9 buffer	Abcam	Cat#ab93684
Bovine Serum Albumin Fraction V	Fisher Scientific	Cat#BP1605-100
R-Phycoerythrin lightning-link labeling kit	Novus Biologicals	Cat#703-0010
Alexa 647 labeling kit	Thermofisher	Cat# A20186
PHEM buffer	Goldbio	https://www.goldbio.com/documents/3553/PHEM+Buffer+4X+Stock+Solution.pdf
DAPI	Biolegend	Cat#422801
ProLong Gold antifade mounting media	Thermofisher	Cat#P36934

**Critical commercial assays**		

GeoMx Cancer Transcriptome Atlas (COVID-19)	Nanostring	https://www.nanostring.com/products/geomx-digital-spatial-profiler/geomx-rna-assays/geomx-cancer-transcriptome-atlas/

**Deposited data**		

Transcriptomics data	This paper	https://doi.org/10.17632/n5dn4xzg7j.1

**Software and algorithms**		

Prism v7	GraphPad	https://www.graphpad.com/scientific-software/prism/
R v3.5.2	R project	https://www.r-project.org/
Microsoft Excel	Microsoft	https://www.microsoft.com/en-us/microsoft-365/p/excel/cfq7ttc0k7dx?activetab=pivot:overviewtab
Adobe Illustrator 2020	Adobe	https://www.adobe.com/products/illustrator.html
GSEA	Broad Insititute	https://www.gsea-msigdb.org/gsea/index.jsp
GeoMx DSP data center	Nanostring	https://www.nanostring.com/products/geomx-digital-spatial-profiler/geomx-data-center/

### Resource availability

#### Lead contact

Further information and requests for resources and reagents should be directed to and will be fulfilled by the Lead Contact, Dr. Amit Gaggar (agaggar@uabmc.edu).

#### Materials availability

This study did not generate new unique reagents.

#### Data and code availability

The datasets generated during this study are available on Mendeley: https://doi.org/10.17632/n5dn4xzg7j.1.

### Experimental model and subject details

#### Human subjects

Pulmonary autopsy specimens were collected from patients deceased due to ARDS. Three patients were infected with SARS-CoV-2, three patients were infected with influenza A subtype H1N1, and one patient was infected with both SARS-CoV-2 and H1N1. The study was approved by the Institutional Review Board (UAB-IRB 300005258, VA-IRB 1573682) and summary demographic and clinical data are presented in Table S1.

### Method details

#### Histology

Lungs were inflated isobarically with 10% formalin and preserved in paraffin blocks. Sequential tissue sections of 5μm were used for viral staining by immunofluorescence, for RNA analysis, and for hematoxylin and eosin or Masson’s trichrome staining to identify ARDS pathological features and collagen deposition respectively.

#### Immunofluorescence

Paraffin-embedded tissue slides were incubated for 2 hours at 60°C. Deparaffinization and rehydration of the slides was performed with sequential three 5 minutes incubations in xylene (Fisher Scientific), two sequential 5 minutes incubations in 100% denatured ethyl alcohol (Fisher Scientific), two sequential 5 minutes incubations in 95% denatured ethyl alcohol (Fisher Scientific), followed by three sequential 5 minutes incubations in distilled water under 40rpm gentle shaking. Antigen retrieval was performed in pre-warmed Tris-EDTA pH 9 buffer (Abcam) at 70°C in heated steamer for 20 minutes, followed by three washes in distilled water under 40rpm gentle shaking. Tissue sections were blot dry and incubated with PBS for 10 minutes at room temperature, followed by blockade of non-specific binding using 3% w/v BSA (Fisher Scientific) for 40 minutes at room temperature. The SARS-CoV-2 only infected slides and the SARS-CoV-2/H1N1 slide were stained for 1 hour at room temperature with anti-SARS-CoV-2 nucleocapsid antibody (GeneTex, GTX135361, RRID: AB_2887484) directly labeled with R-Phycoerythrin lightning-link labeling kit (Novus Biologicals) at 1:500 dilution in PBS, 3% w/v BSA. Slides were then washed three times for 5 minutes in PBS under gentle agitation.

The SARS-CoV-2/H1N1 slide and the H1N1 only infected slides were then fixed for 10 minutes at room temperature with 4% paraformaldehyde and washed three times with PBS, 0.1% Tween-20 (PBST) for 5 minutes. Staining for H1N1 was performed with Alexa 647 (Thermofisher) pre-labeled anti-influenza A virus nucleoprotein antibody (Abcam, ab20343, RRID:AB_445525) at 1:200 in PHEM buffer (Goldbio) for 1 hour at room temperature. Tissues were then washed three times in PBST for 5 minutes.

Staining for EpCAM (Abcam, ab213500, clone: EPR20532-222, 1:200 in 3% BSA), CD68 (Abcam, ab224029, clone: EPR20545, 1:1000 in PHEM buffer), alpha-smooth muscle actin (Invitrogen, 53-9760-82, clone:1A4, RRID: AB_2574461 1:400 in PHEM buffer), KRT19 (Thermofisher, TA803863, clone:OTI6A8, 1: 1:500 in 3% BSA), TGFBR (Thermofisher, TA807912, clone:OTI3B4, 1:500 in PHEM buffer), and F13A1 (Abcam, ab225018, clone:EP3372, 1:500 in PHEM buffer) was performed for 1 hour at room temperature and then washed three times for 5 minutes in PBST under gentle agitation.

Nuclei counter staining for all slides was performed using 300nM DAPI (Biolegend) in PBS for 5 minutes, followed by three 5 minutes washes in PBS. Slides were mounted using ProLong Gold antifade mounting media with DAPI (Thermofisher) and stored in the dark until image acquisition. Confocal immunofluorescence images were acquired using the Nikon A1R confocal microscope.

#### GeoMX digital spatial profiling

Paraffin embedded tissues were processed and analyzed at NanoString techonology laboratories using a combination of fluorescently labeled antibodies, anti-CD68 (Santa Cruz, sc-20060 AF647, clone: KP1, RRID: AB_627158), anti-EpCAM (Abcam, ab213500, clone:EPR20532-222), anti-smooth muscle actin (Invitrogen, 53-9760-82, clone:1A4, RRID: AB_2574461) and the GeoMX COVID-19 Immune Response Atlas gene set with custom probe set specific for SARS-CoV-2 lung infection and tissue responses (see Table S2 for SARS-CoV-2 related gene list and Figure S1 for workflow), totaling 1860 genes. Selection of regions of interests (ROI, 12 per patient) was performed based on the immunofluorescent viral staining, the cellular immunofluorescent profile and the pathological features of ARDS (i.e., presence of hyaline membranes and diffused alveolar damage) observed in the H&E stained sections. To ensure even and representative selection of ROIs, lower left lobe of the lung was analyzed in all subjects. Each patient had 2-4 total lung areas selected in regions of ARDS (confirmed by pathologist), 2-4 ROIs for epithelial cells (normal epithelium versus hyperplastic), 2-3 vascular beds selected, and 2-4 macrophage populations (infiltrate and clusters). For cell-specific profiling (Figures 2, 3, and 4), at least 50 cells per ROI were utilized for analyses.

### Quantification and statistical analysis

Sequencing through the Nanostring GeoMx platform is performed on the RNA probe tag and not on the transcript itself, providing less sequencing bias and a more accurate transcript count. RNA probe counts used in the analyses were selected following a sequencing QC according to Nanostring protocols, where counts from each area of interest are analyzed and under-sequenced samples are dropped (field of view percentage of 75% and Binding density from 0.1 to 2.25), and a probe QC, where mRNAs are targeted by multiple probes and outlier probes are dropped from downstream data analysis (positive spike-in normalization factor between 0.3 and 3). Then RNA counts were normalized using a signal-based normalization, in which individual counts are normalized against the 75^th^ percentile of signal from their own area of interest. The final list of detectable genes was then obtained by dropping genes in each specific group (ARDS regions, vascular, epithelium, macrophages) by using a limit of quantification (LOQ) of 20% coverage within replicates. The LOQ was calculated using the geometric mean and geometric standard deviation of negative probes in the dataset. Counts were normalized to log2 and statistical comparisons were performed using a two sample t test upon normality testing and ComBat correction for batch effect^40^^,^^41^. Comparison of SARS-CoV-2 to H1N1 was performed by averaging the technical replicates and by comparing biological replicates (n = 3 per group). Comparison of the double infected patient to the single infection was done using technical replicates (ROIs) as unique samples for statistical reasons. P value threshold for differential gene expression were set at p = 0.02 and log2 fold change of 0.5. All details for the statistical analyses and number of replicates can be found in the figure legends. All analyses for the volcano plots can be found in the Data S1 file.

Statistical analysis done for specific gene expression (Figure S4) was performed using Mann-Whitney test and data are shown as median and interquartile range (n = 3 per group).

## Supplemental information

Document S1. Figures S1–S6 and Tables S1 and S2

Data S1. Summary of gene-expression analysis used in volcano plots, related to STAR Methods

Document S2. Article plus supplemental information
