# Supplementary material for: Spatial mapping of SARS-CoV-2 and H1N1 lung injury identifies differential transcriptional signatures
Source: Cell Rep Med. 2021 Mar 23;2(4):100242. doi: 10.1016/j.xcrm.2021.100242 (PMC7985929; doi:10.1016/j.xcrm.2021.100242)
Supplement: Document S1. Figures S1–S6 and Tables S1 and S2 [file mmc1.pdf]

Cell Reports Medicine, Volume *2*

Supplemental information

Spatial mapping of SARS-CoV-2 and H1N1 lung injury identifies differential transcriptional signatures

Camilla Margaroli, Paul Benson, Nirmal S. Sharma, Matthew C. Madison, Sarah W. Robison, Nitin Arora, Kathy Ton, Yan Liang, Liang Zhang, Rakesh P. Patel, and Amit Gaggar

Supplementary Materials for

Spatial mapping of SARS-CoV-2 and H1N1 Influenza Lung Injury Identifies Differential Transcriptional Signatures

C. Margaroli, P. Benson, N.S Sharma, M.C. Madison, S.W. Robison, N. Arora, K. Ton, Y. Liang, L. Zhang, R.P. Patel, A. Gaggar

Correspondence to: agaggar@uabmc.edu

This PDF file includes:

Figs. S1 to S6
Tables S1 to S2

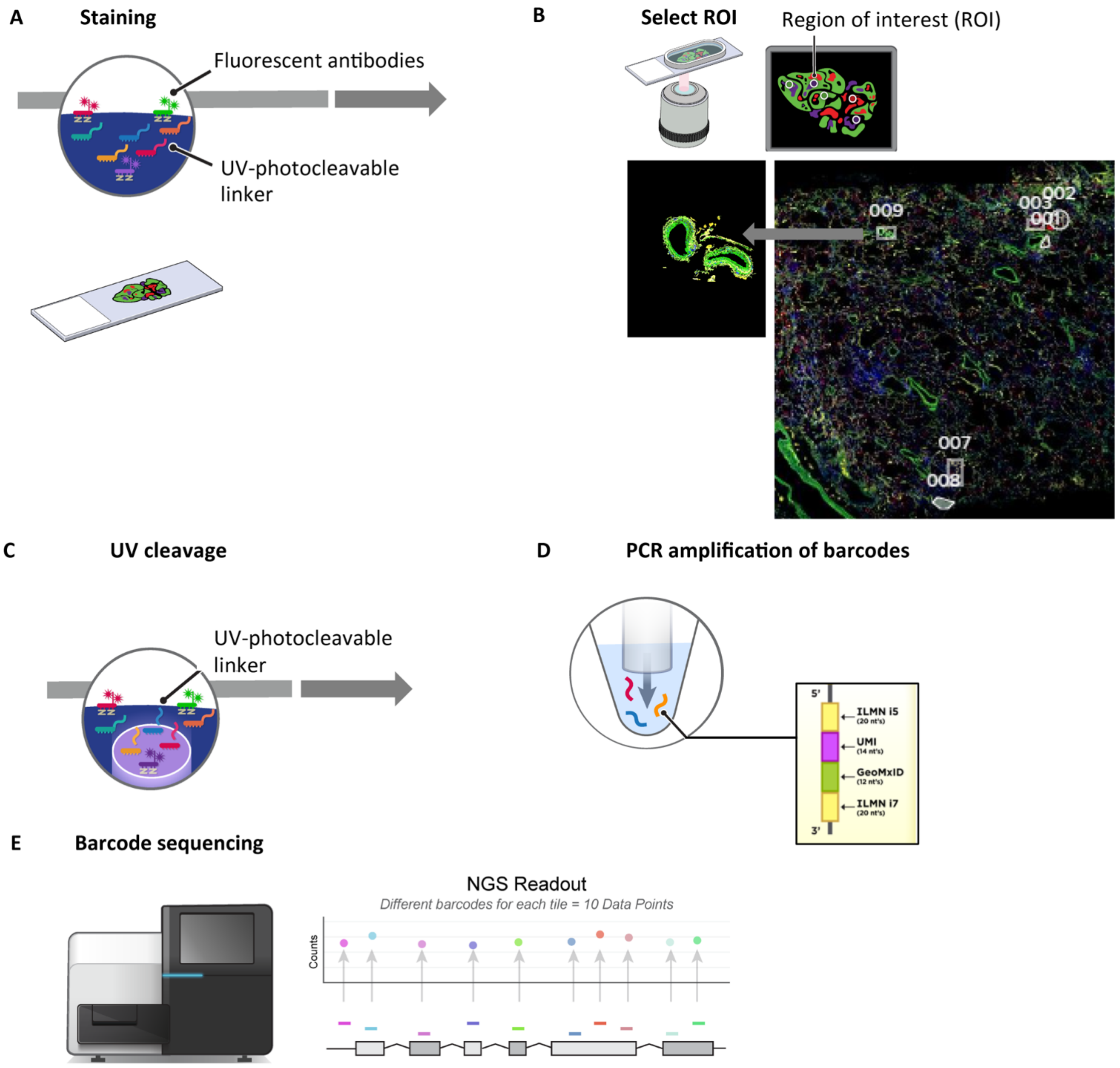

Fig. S1 related to STAR Methods. Nanostring workflow. (A) The tissue is processed and stained using a standard ISH protocol with both fluorescent labeled morphology markers and sequence specific RNA probes. **(B)** Morphology markers are used for Region of Interest (ROI) and Area of Interest (AOI) selection. **(C)** After ROI/AOI selection, UV light is shone on the ROI/AOI decoupling the barcodes from the RNA probes only from the selected area or cells of interest. Five probes per mRNA target are used and are coupled to molecular barcodes and a unique barcode identifies a specific gene target. The UMI identifies a specific molecule and accounts for amplification bias from PCR. **(D)** RNA probes for the ROI/AOI are aspirated and deposited into a unique well of a 96 well plate. The PCR step adds dual indexing barcodes (Index1 and Index2) to identify AOIs and adds Illumina flow cell adapter regions (P5 and P7) to enable sequencing. **(E)** The barcodes are sequenced.

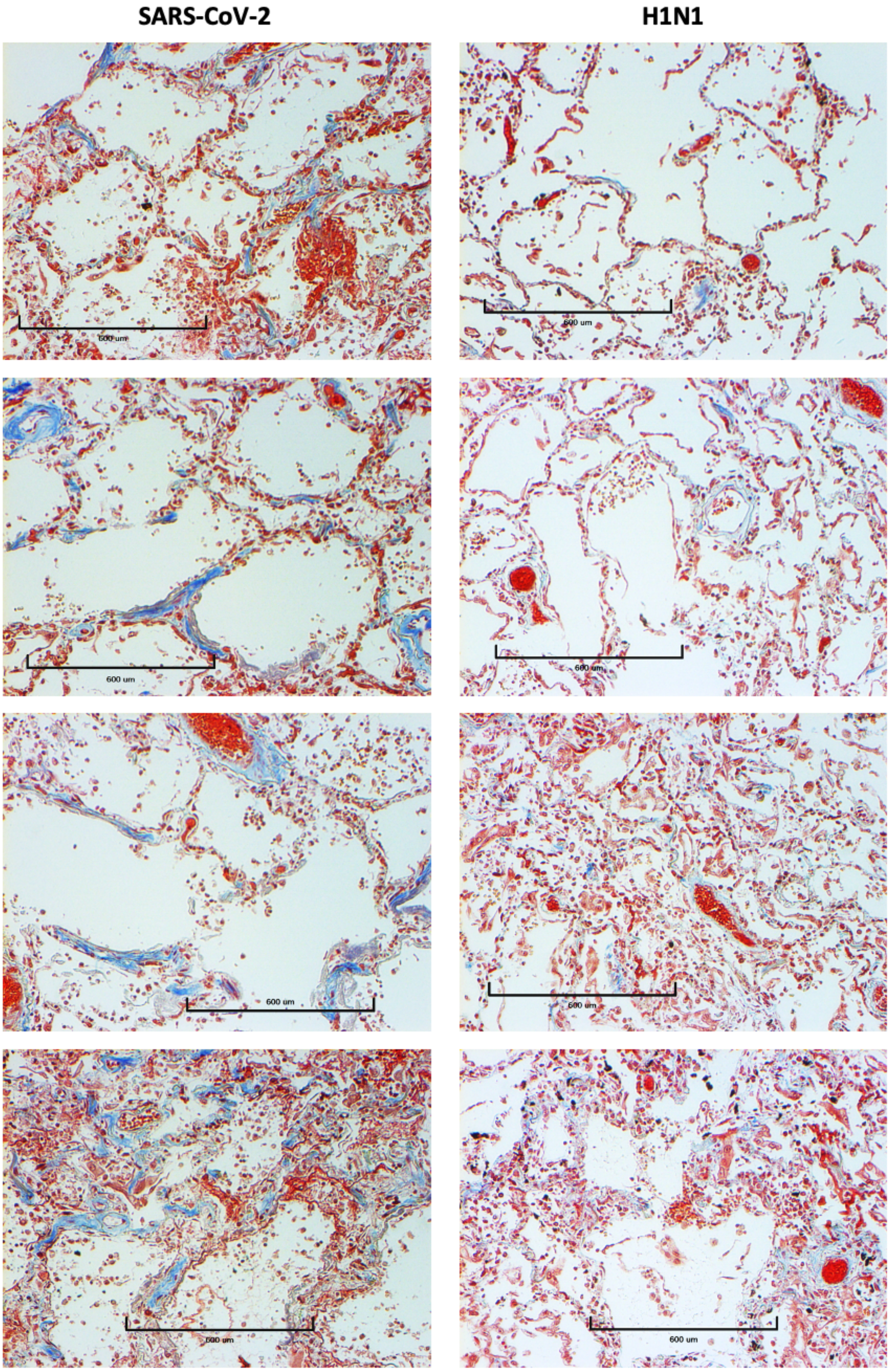

Fig. S2 related to Figure 1. Representative areas of collagen deposition in H1N1 and SARS-CoV-2 lung tissue. Collagen staining (blue) was performed by Masson's trichrome staining on paraffin embedded lung tissues. Scale bar 600μm. SARS-CoV-2 infected patients (n=3), H1N1 (n=3).

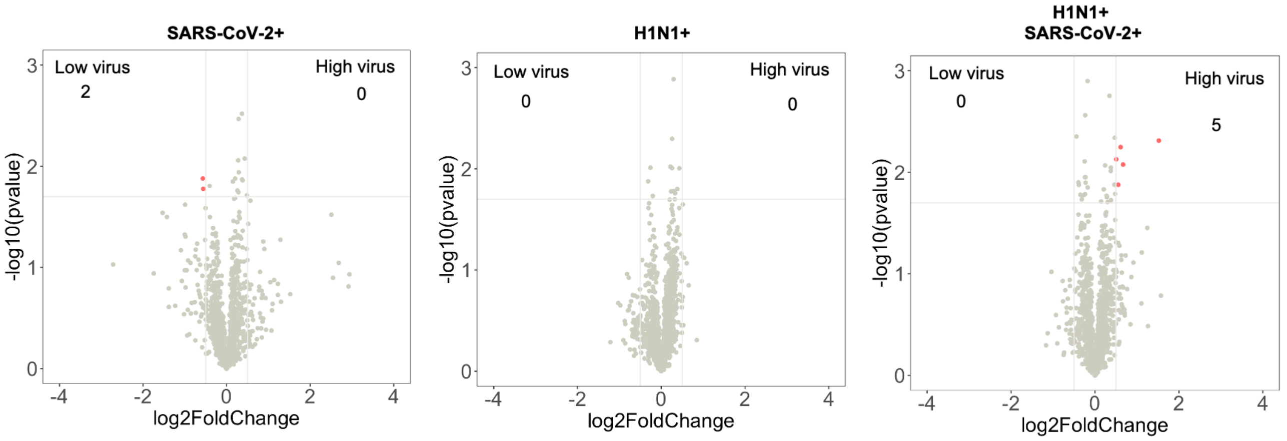

Fig. S3 related to Figure 1. Viral load does not influence gene expression. Differential gene expression comparing areas of high and low viral presence determined by immunofluorescence in patients infected by SARS-CoV-2, H1N1 or both viruses, showed no differential modulation of gene expression. P-value threshold for differential gene expression were set at p=0.02 and log2 fold change of 0.5. Biological replicates were analyzed for SARS-CoV-2 infected patients (n=3) and H1N1 (n=3), technical replicates were used for SARS-CoV-2/H1N1 (high n=2, low n=2).

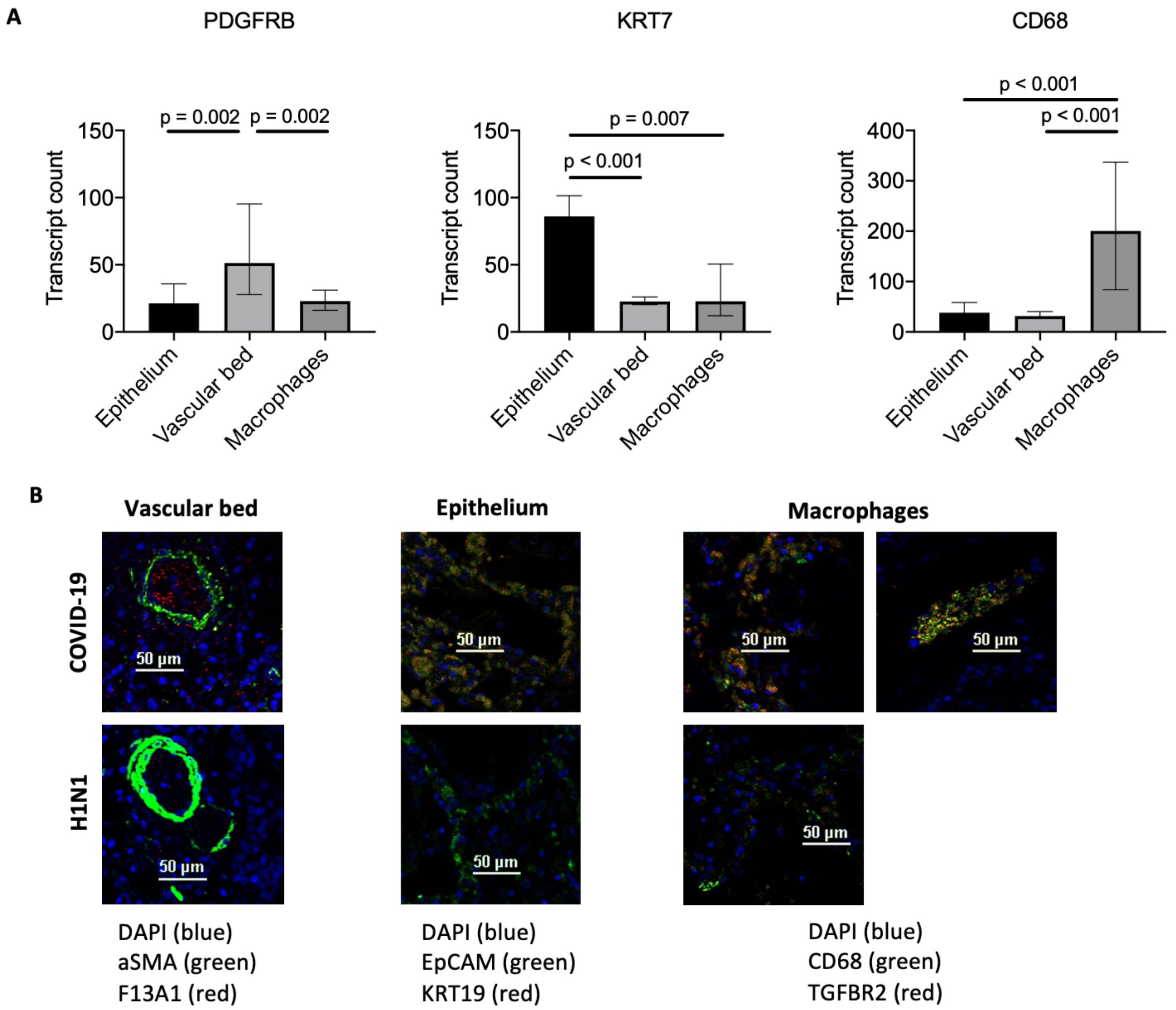

Fig. S4 related to Figures 2, 3, and 4. Gene and protein expression in cell populations selected by immunofluorescence. Specific cell populations for transcriptional profiling were selected using immunofluorescence staining. **(A)** Validation of cellular purity was performed by gene expression in the selected samples, for vascular bed (PDGFRB), alveolar epithelium (KRT7), and macrophages (CD68). Replicates for expression include SARS-CoV-2 infected patients (n=3), H1N1 (n=3), and SARS-CoV-2/H1N1 (n=1). **(B)** Validation of colocalization for high expressed genes in SARS-CoV-2 tissue for epithelium, vascular bed and macrophages. Scale bars= 50μm. Results are shown as median and interquartile range. Data were analyzed using Mann-Whitney test.

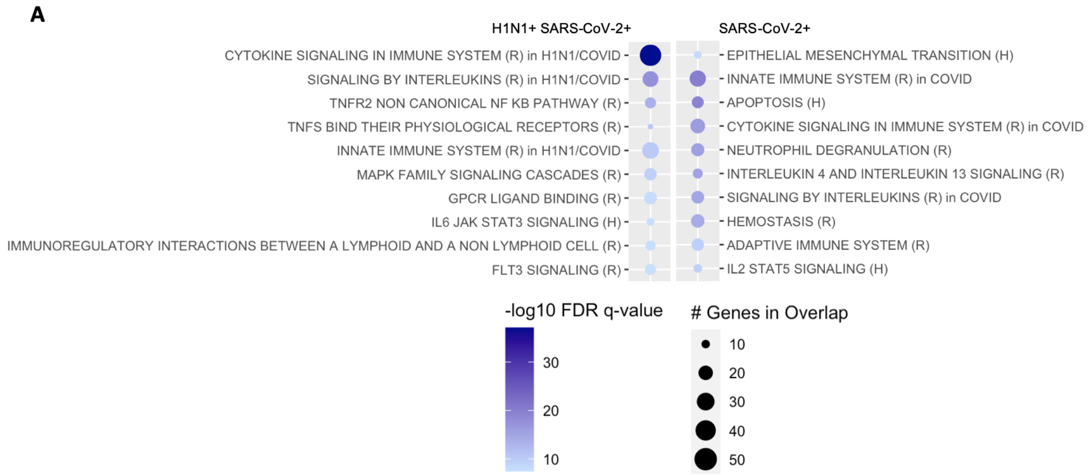

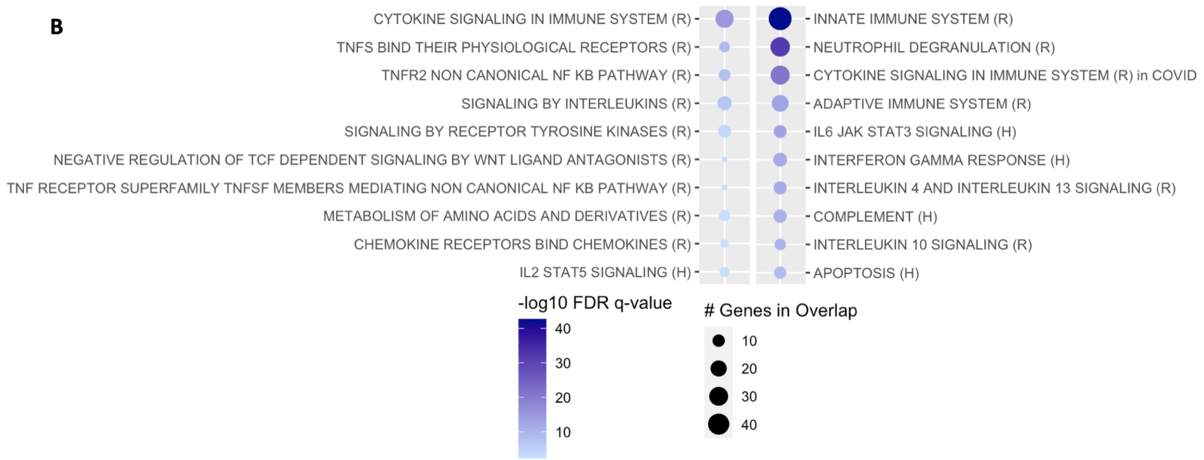

Fig. S5 related to Figures 3 and 4. SARS-CoV-2/H1N1 infected patient mimic H1N1 only gene expression. Geneset enrichment analysis using reactome (R) and hallmark (H) datasets for up-regulated or down-regulated genes in SARS-CoV-2 infected patients compared to SARS-CoV-2/H1N1 sample set for the epithelium **(A)** (technical replicates n= 12 for SARS-CoV-2 and n=5 SARS-CoV-2/H1N1), and macrophages **(B)** (technical replicates n= 9 for SARS-CoV-2 and n=3 SARS-CoV-2/H1N1). Differential gene expression was defined as p-value of 0.02 and log2 fold change of 0.5. SARS-CoV-2 infected patients (n=3) and SARS-CoV-2/H1N1 (n=1).

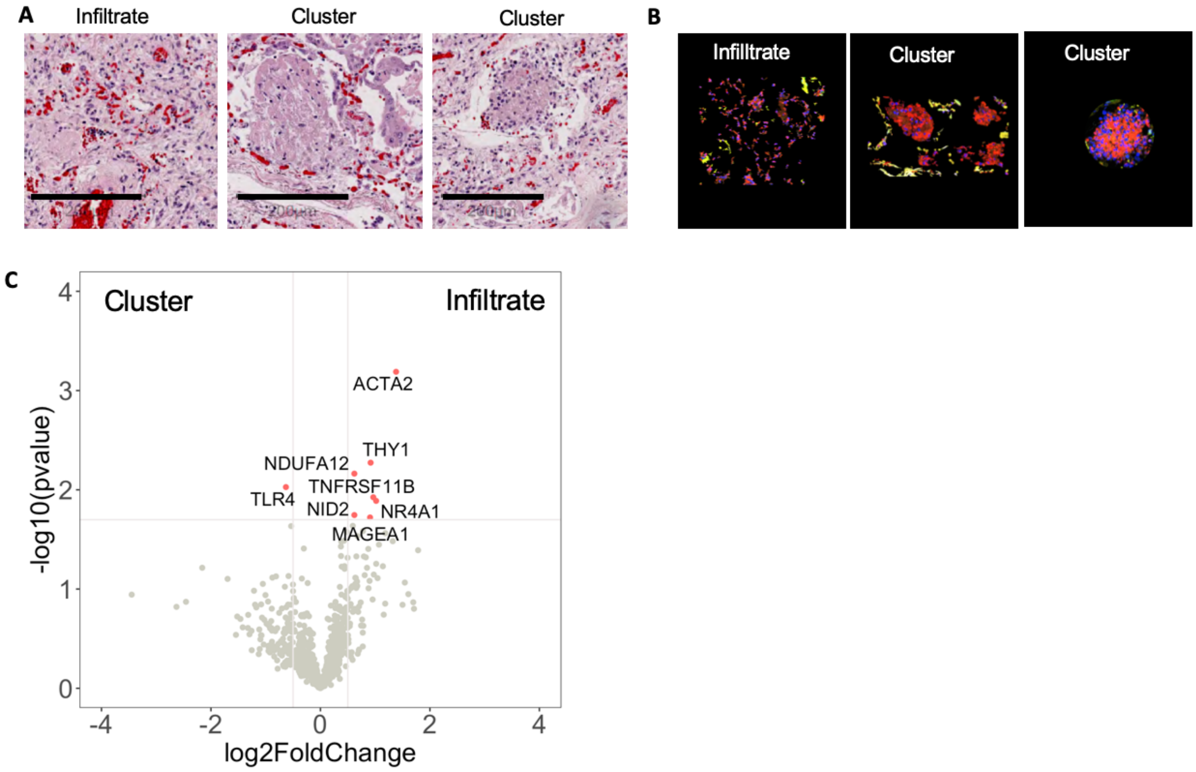

Fig. S6 related to Figure 4. Analysis of clustering macrophage transcriptional phenotype in SARS-CoV-2 patients. **(A)** Histological analysis of tissues sections stained by H&E revealed presence of clustering macrophages (scale bars = 200μm), which was confirmed by **(B)** CD68 staining (Blue = nuclei, Red = CD68, Yellow = EpCAM). Differential gene expression analysis of clustering vs infiltrating macrophages in SARS-CoV-2 infected patients showed only 8 differentially regulated genes. Differential gene expression was defined as p-value of 0.02 and log2 fold change of 0.5. SARS-CoV-2 infected patients (n=2). Technical replicates n= 4 for infiltrate and n=4 for cluster.

	Patient 1	Patient 2	Patient 3	Patient 4	Patient 5	Patient 6	Patient 7
**VIRAL TESTING**							
SARS-CoV-2	Positive	Positive	Positive	Negative	Negative	Negative	Positive
Influenza- H1N1	Negative	Negative	Negative	Positive	Positive	Positive	Positive
**DEMOGRAPHICS**							
Age in years	86	69	75	57	50	63	58
Race	C	AA	C	C	C	AA	AA
Gender	Male	Male	Male	Female	Female	Male	Female
**CO-MORBIDITIES**							
Diabetes Mellitus	None	Yes	None	None	None	None	None
CV Disease	None	None	None	None	HFpEF	None	None
Chronic Lung Disease	Asthma	None	None	None	COPD	None	CTD-ILD, COPD
Chronic Kidney Disease	None	None	None	None	None	None	None
**INTERVENTIONS**							
Antiviral (days)	None	None	Remdesivir (2)	Oseltamivir (7)	Oseltamivir (7)	Oseltamivir (10)	Oseltamivir (10)
Steroids (days)	MP (3)	HC (16)	Dex (1)	HC (3)	MP (2)	Pred (3)	HC (6)
Epoprostenol (days)	None	None	EPO 2d	None	EPO (16)	NO, EPO (3)	EPO (4)
Mechanical Ventilation	100% FiO2 via NRB	5.7cc/kg Tv, 12 PEEP, 60% FiO2	5.3cc/kg Tv, 16 PEEP, 60%FiO2	6cc/kg Tv, 16 PEEP, 100% FiO2	5.3cc/kg Tv, 18 PEEP, 100% FiO2	5.1cc/kg Tv, 15 PEEP, 90% FiO2	5.9cc/kg Tv, 10 PEEP, 100% FiO2
Proning (days)	None	Yes (9)	Yes (3)	None	Yes (5)	Yes (4)	None
ECMO (days)	None	None	None	VA (5)	None	None	None
**ICU ADMISSION**							
BMI (kg/m^2)	22.6	28	32.3	30	68.9	27.5	31
WBC (x10^3/cmm)	14.8	12.7	8.14	0.9	6.35	11.92	11.6
% PMN	81%	90%	88%	4%	87%	86%	84%
% Lymphocytes	6%	6%	7%	72%	7%	5%	9%
Troponin I (ng/L)	<0.3	88	17	38	128	n/a	1761
BNP (pg/mL)	n/a	78	536	445	581	n/a	299
AST (U/L)	34	106	68	15	41	32	43
Creatinine (mg/dL)	1.9	1.6	1.1	0.9	1.5	1.4	1.0
D-Dimer (ng/mL)	n/a	>20,000	921	2,978	n/a	n/a	12,301
PT (sec)	16.2	14.6	13.4	15.1	16.5	13.1	13.2
LDH (U/L)	n/a	777	n/a	198	n/a	n/a	472
**PHYSIOLOGY**							
Chest Radiograph (# of quadrants [1-4])	4	2	4	2	4	4	4
PaO2:FiO2 ratio	61	120	173	64	47	132	113
APACHE II Score (a) admission (b) 48h before death	(a) 20 (b) 24	(a) 26 (b) 35	(a) 20 (b) 43	(a) 17 (b) 24	(a) 13 (b) 37	(a) 20 (b) 38	(a) 14 (b) 17
SOFA Score (a) admission (b) 48h before death	(a) 5 (b) 6	(a) 12 (b) 18	(a) 10 (b) 17	(a) 10 (b) 17	(a) 11 (b) 14	(a) 7 (b) 15	(a) 7 (b) 7
**30-DAY**							
ICU Days	5	16	4	6	19	11	10
Ventilator Days	0	16	4	6	17	8	8
New Organ Failure	3	5	3	5	2	3	3

Table S1 related to STAR methods. Summary of patient demographics. Abbreviations: C: Caucasian; AA: African American; CV: cardiovascular; HFpEF: heart failure preserved ejection fraction; CTD-ILD: connective tissue disease associated interstitial lung disease; COPD: chronic obstructive lung disease; MP: Methylprednisone; HC: Hydrocortisone; TV: tidal volume, PEEP: positive end-expiratory pressure, FIO2: fraction of inspired oxygen, ECMO: extracorporeal membrane oxygenation, V-A: venous-arterial; BMI: body mass index; WBC: white blood cells; BNP: brain natriuretic

peptide; AST: aspartate aminotransferase; PT: prothrombin time; LDH: lactic acid dehydrogenase; PaO2: partial pressure arterial oxygen; APACHE: acute physiology and chronic health evaluation; SOFA: sequential organ failure assessment; ICU: intensive care unit.

COVID-19 added gene set

S	Spike Protein
ORF1ab	ORF1ab
ORF1ab_REV	Negative Strand ORF1ab
ACE2	angiotensin I converting enzyme 2
ABCA3	ATP binding cassette subfamily A member 3
AQP5	aquaporin 5
DHX58	DEXH (Asp-Glu-X-His) box polypeptide 58
FURIN	furin (paired basic amino acid cleaving enzyme)
HAS2	hyaluronan synthase 2
HOPX	HOP homeobox
IFNLR1	interferon lambda receptor 1
IL10RB	interleukin 10 receptor subunit beta
MUC13	mucin 13, cell surface associated
MUC2	mucin 2, oligomeric mucus/gel-forming
MUC5AC	mucin 5AC, oligomeric mucus/gel-forming
MUC5B	mucin 5B, oligomeric mucus/gel-forming
NAPSA	napsin A aspartic peptidase
PGC	progastricsin (pepsinogen C)
SCGB1A1	secretoglobin family 1A member 1
SFTPA1	surfactant protein A1
SFTPB	surfactant protein B
SFTPC	surfactant protein C
SFTPD	surfactant protein D
TP63	tumor protein p63
CC2D1B	coiled-coil and C2 domain containing 1B
SF3A3	splicing factor 3a subunit 3

Table S2 related to STAR methods. GeoMx spatial profiling SARS-CoV-2 gene set.
