# Supplementary material for: Spatial mapping of SARS-CoV-2 and H1N1 lung injury identifies differential transcriptional signatures
Source: Cell Rep Med. 2021 Mar 23;2(4):100242. doi: 10.1016/j.xcrm.2021.100242 (PMC7985929; doi:10.1016/j.xcrm.2021.100242)
Supplement: Document S2. Article plus supplemental information [file mmc3.pdf]

Cell Reports
Medicine

Report

Spatial mapping of SARS-CoV-2 and H1N1 lung injury identifies differential transcriptional signatures

Graphical abstract

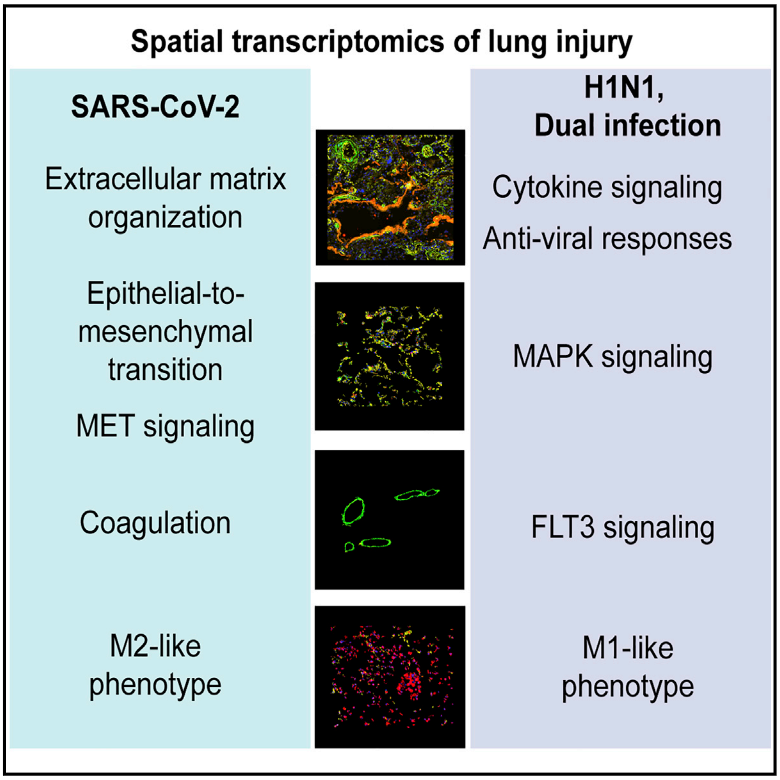

Authors

Camilla Margaroli, Paul Benson, Nirmal S. Sharma, ..., Liang Zhang, Rakesh P. Patel, Amit Gaggar

Correspondence

agaggar@uabmc.edu

In brief

Lung injury can be induced by viral infections, such as H1N1 and SARS-CoV-2 infections. Here, Margaroli et al. investigate differential lung responses to both viruses. SARS-CoV-2-induced genes relate to tissue remodeling, while the H1N1 profile is skewed toward anti-viral responses.

Highlights

- SARS-CoV-2 and H1N1-induced ARDS show unique transcriptional signatures
- Tissue remodeling pathways dominate the transcriptional profile of SARS-CoV-2 ARDS
- SARS-CoV-2/H1N1 double infection resembles the lung anti-viral response to H1N1

Margaroli et al., 2021, Cell Reports Medicine 2, 100242
April 20, 2021 © 2021 The Author(s).
https://doi.org/10.1016/j.xcrm.2021.100242

CellPress

Cell Reports Medicine

CellPress OPEN ACCESS

Report

Spatial mapping of SARS-CoV-2 and H1N1 lung injury identifies differential transcriptional signatures

Camilla Margaroli,[1,2] Paul Benson,[3] Nirmal S. Sharma,[2,4] Matthew C. Madison,[1,2] Sarah W. Robison,[1,2] Nitin Arora,[5] Kathy Ton,[6] Yan Liang,[6] Liang Zhang,[6] Rakesh P. Patel,[2,7,8] and Amit Gaggar[1,2,8,9,10,*]

[1]Department of Medicine, Division of Pulmonary, Allergy & Critical Care Medicine, University of Alabama at Birmingham, Birmingham, AL, USA

[2]Program in Protease/Matrix Biology, University of Alabama at Birmingham, Birmingham, AL, USA

[3]Department of Pathology, Division of Anatomic Pathology, University of Alabama at Birmingham, Birmingham, AL, USA

[4]Department of Medicine, Division of Pulmonary, Allergy, and Critical Care Medicine, Brigham and Women's Hospital, Boston, MA, USA

[5]Department of Pediatrics, Division of Neonatology University of Alabama at Birmingham and Children's Hospital of Alabama, Birmingham, AL, USA

[6]Nanostring Technologies Inc., Seattle, WA, USA

[7]Department of Pathology, Division of Molecular & Cellular Pathology, University of Alabama at Birmingham, Birmingham, AL, USA

[8]Center for Free Radical Biology, University of Alabama at Birmingham, Birmingham, AL, USA

[9]Birmingham VA Medical Center, Birmingham, AL, USA

[10]Lead contact

*Correspondence: agaggar@uabmc.edu

https://doi.org/10.1016/j.xcrm.2021.100242

SUMMARY

Severe SARS-CoV-2 infection often leads to the development of acute respiratory distress syndrome (ARDS), with profound pulmonary patho-histological changes post-mortem. It is not clear whether ARDS from SARS-CoV-2 is similar to that observed in influenza H1N1, another common viral cause of lung injury. Here, we analyze specific ARDS regions of interest utilizing a spatial transcriptomic platform on autopsy-derived lung tissue from patients with SARS-CoV-2 (n = 3), H1N1 (n = 3), and a dual infected individual (n = 1). Enhanced gene signatures in alveolar epithelium, vascular tissue, and lung macrophages identify not only increased regional coagulopathy but also increased extracellular remodeling, alternative macrophage activation, and squamous metaplasia of type II pneumocytes in SARS-CoV-2. Both the H1N1 and dual-infected transcriptome demonstrated an enhanced antiviral response compared to SARS-CoV-2. Our results uncover regional transcriptional changes related to tissue damage/remodeling, altered cellular phenotype, and vascular injury active in SARS-CoV-2 and present therapeutic targets for COVID-19-related ARDS.

INTRODUCTION

Acute respiratory distress syndrome (ARDS) has been observed following respiratory viral infections, most notably during H1N1 influenza pandemics[1] and during the current COVID-19 pandemic caused by the the severe acute respiratory syndrome-coronavirus 2 (SARS-CoV-2).[2,3] Both SARS-CoV-2 and H1N1-mediated ARDS have been characterized by increased lung inflammation and increased disease-related morbidity and mortality.[4–6] However, there has been recent evidence suggesting that SARS-CoV-2 patients have extended hospitalizations in subjects with ARDS compared to influenza-induced ARDS,[7] indicating that the cellular processes that drive this pathology may differ between these two important viral causes of lung injury.

Post-mortem lung and vascular tissues from SARS-CoV-2 subjects have shown profound morphological changes,[8,9] with diffuse alveolar damage, induction of fibrotic responses in the lung epithelium,[10] and presence of vascular congestion and thrombi.[8,11] Furthermore, temporal and spatial heterogeneity of lung responses to SARS-CoV-2 have been reported,[12] including differential expression of interferon gamma in patients with high viral load. However, the transcriptional alterations identified while preserving the tissue architecture and within the lungs of SARS-CoV-2 ARDS subjects compared to other viral forms of ARDS remain poorly defined. There are also limited data related to potential co-infection of both viruses leading to ARDS,[13] as there has been little influenza during the current fall/winter period. Here, we analyzed the histological and transcriptional response of key structural and immune cells, while preserving the tissue architecture of the lung, in ARDS patients infected with SARS-CoV-2, H1N1, and an individual who was infected with both viruses.

RESULTS

COVID-19 ARDS lung has a discrete regional transcriptomic profile

To investigate similarities and regional differences in viral-induced ARDS, we used autopsy lung tissue from seven patients

This is an open access article under the CC BY license (http://creativecommons.org/licenses/by/4.0/).

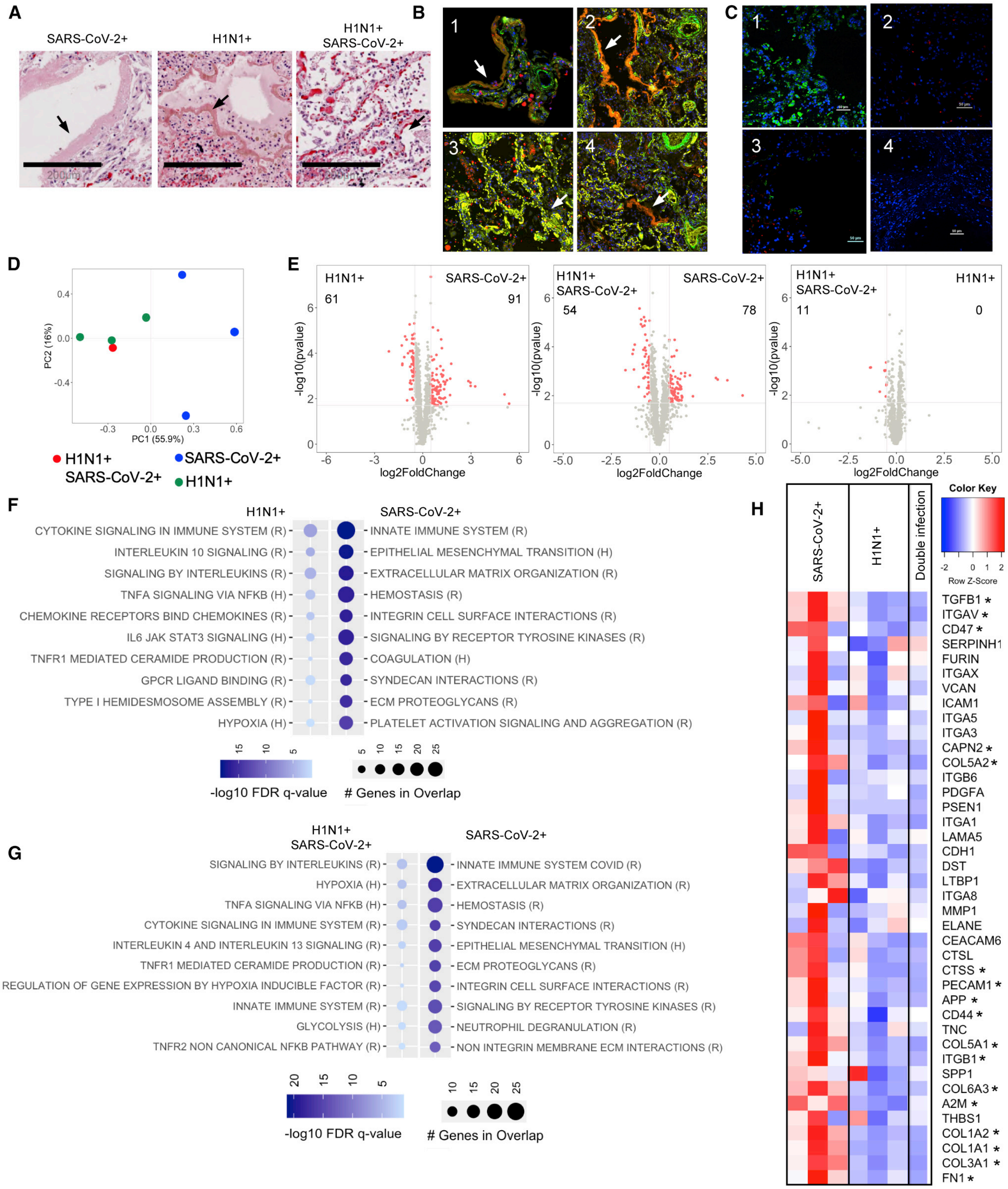

Figure 1. SARS-CoV-2-induced lung injury shows a discrete transcriptional signature

(A) Histological analysis of tissues sections stained by H&E (scale bars, 200 μm) revealed presence of ARDS in all three patient groups; arrows indicate hyaline membranes.

(B) Immunofluorescent staining (10× magnification) of α-SMA (green), CD68 (red), and EpCAM (yellow) in SARS-CoV-2 (1), H1N1 (2), double infected (3), and areas of low viral load (4); arrows indicate hyaline membranes.

(legend continued on next page)

CellPress
OPEN ACCESS

diagnosed with ARDS and confirmed SARS-CoV-2 or H1N1 infection (Table S1). We leveraged the GeoMX Digital Spatial Profiling platform to delineate and sequence-specific regions of interest (ROIs) from tissues, *in situ* hybridized with an 1860 gene platform (GeoMX COVID-19 Immune Response Atlas) (STAR Methods; Figure S1).

All patients displayed histologic regions of ARDS defined as diffused alveolar damage (DAD) and by the presence of alveolar epithelial injury (hyaline membranes) (Figure 1A).[14] These regions were subsequently stained by immunofluorescence to delineate epithelial ($EpCAM^+$), vascular (smooth muscle $actin^+$), and macrophage populations ($CD68^+$) (Figure 1B) and selected for transcriptional profiling. Further, these regions were stained with antibodies directed to SARS-CoV-2 or influenza A to identify areas of increased versus low viral burden (Figure 1C).

The selected ARDS regions, which delineated the transcriptional response of the total lung tissue, demonstrated differences in transcriptome expression between the SARS-CoV-2 versus both H1N1 alone and dual viral infection regions (Figures 1D and 1E). Although immune activation pathways were found to predominate in the H1N1 (Figure 1F) and dual-infected regions (Figure 1G), SARS CoV-2 regions demonstrated increased expression of epithelial-to-mesenchymal transition (EMT), coagulation, and extracellular matrix (ECM) pathways. This notable increase in ECM-related genes in SARS-CoV-2 included increased ECM components, ECM-related signaling molecules, and proteases (Figure 1H), which resulted in presence of higher collagen deposition in the SARS-CoV-2 lung tissue compared to H1N1 (Figure S2). Interestingly, the gene expression of virus-rich versus virus-poor regions within each infection group did not show significant differences (Figure S3).

To further identify transcriptional signatures of structural lung cells and resident immune cells that may contribute to the ARDS phenotype, cells of the vascular bed (smooth muscle $actin^+$), epithelium ($EpCAM^+$), and macrophage populations ($CD68^+$) were selected by immunofluorescent staining. The purity of the selected populations and their RNA was validated by the expression of cell-specific genes (Figure S4A): PDGFRB (vascular),[15] KRT7 (epithelium),[16] and CD68 (macrophages),[17] which followed the expected trends. Furthermore, using immunofluorescence we assessed protein expression of one of the most upregulated genes in SARS-CoV-2 patients compared to H1N1 in each cell type (Figure S4B), showing co-localization of the protein with the selected cell type.

COVID-19 ARDS-associated vascular beds show increased hypercoagulopathy

Due in part to recent evidence of hypercoagulopathy in SARS-CoV-2 subjects,[18] we examined vascular beds proximal to regions of DAD (Figures 2A and 2B). Although H1N1 and dual-infected vascular regions demonstrated relatively similar gene expression (Figure 2C, right panel), there were notable differences when these groups were compared to SARS-CoV-2 regions (Figure 2C, left and middle panel). Pathway analyses demonstrated an increased expression of ECM and coagulation-related genes in SARS-CoV-2 versus increased immune signaling in H1N1 and dual-infected vascular cells (Figure 2D; Figure S5A). Genes related to complement activation, platelet biology, and endothelial injury were greatly enhanced in SARS-CoV-2 vascular beds (Figure 2E), demonstrating a transcriptionally regulated response promoting hypercoagulopathy.

Alveolar epithelium in COVID-19 displays squamous metaplasia of type II pneumocytes

We next sought to characterize the alveolar epithelium, a site of notable damage in ARDS (Figures 3A and 3B).[19] As observed with the vascular regions, there was little differential gene expression between dual-infected and H1N1-infected epithelial regions (Figure 3C, right panel). However, there were significant differences in gene expression with SARS-CoV-2 compared to the H1N1 and dual-infected epithelial regions (Figure 3C, left and middle panel). Gene-pathway analyses showed that, although immune signaling was observed in all groups, SARS-CoV-2 regions demonstrated increased EMT and ECM-related pathways (Figure 3D; Figure S5A). Further, we noted that SARS-CoV-2 subjects did demonstrate areas of increased alveolar epithelial hyperplasia (Figure 3E), a phenotype that has recently been described in COVID-19 autopsy specimens.[8] When compared to non-hyperplastic epithelial regions, we observed no significant difference in gene expression (Figure 3F). However, when we compared specific genes related to lung epithelial proliferation and squamous metaplasia of type II pneumocytes in the SARS-CoV-2 hyperplastic and normal epithelium with the non-hyperplastic H1N1 epithelium, we observed increased expression of those genes in the SARS-CoV-2 regions (Figure 3G). These results suggest that cellular metaplasia is an important feature of SARS-CoV-2 ARDS.

Macrophages are differentially activated in COVID-19

As macrophages are critical to the immune response to pulmonary viral infections,[20] we next identified macrophages by H&E and CD68 staining (Figures 4A and 4B). We noted that SARS-CoV-2 patients displayed two phenotypes for pulmonary macrophages: either within clusters or infiltrative phenotype (Figures S6A and S6B). However, when these groups were compared with each other, there were only eight genes that were differentially regulated (Figure S6C) suggesting that these groups are of similar lineage.

(C) Immunofluorescent staining of SARS-CoV-2 (green), H1N1 (red), and DAPI (blue) in SARS-CoV-2 (1), H1N1 (2), double infected (3), and areas of low viral load (4). Scale bars, 50 μm.

(D) PCA analysis of transcriptional signatures in total lung injury.

(E–G) Differential gene-expression analysis and gene set enrichment analysis (GSEA) using reactome (R) and hallmark (H) datasets for upregulated or downregulated genes in SARS-CoV-2-infected patients (n = 3) compared to (F) H1N1 (n = 3) or (G) SARS-CoV-2/H1N1 (n = 1). Differential gene expression was defined as p = 0.02 and log2 fold change of 0.5.

(H) Heatmap representation of genes involved in tissue remodeling and their relative expression in all three types of infection (asterisk indicates significant genes between H1N1 and SARS-CoV-2 shown in the volcano plot).

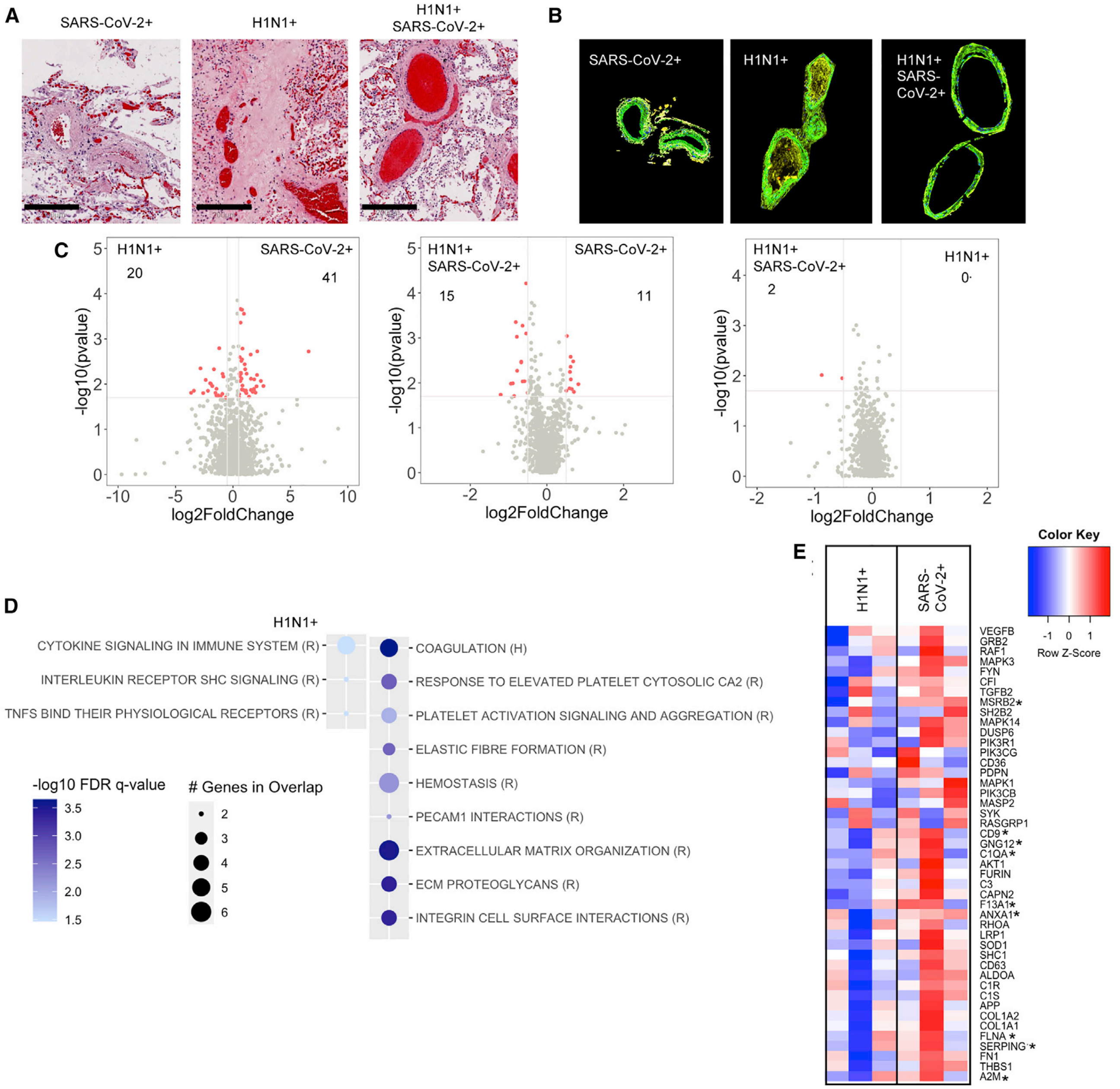

Figure 2. SARS-CoV-2 infection induces a hypercoagulopathy transcriptional program in the pulmonary vascular bed

(A and B) Histological analysis of the lung vascular bed stained (A) by H&E (scale bars, 200 μm) or (B) by immunofluorescence for α-SMA (green).

(C and D) Differential gene-expression analysis (C) and GSEA using reactome (R) and hallmark (H) datasets (D) for upregulated or downregulated genes in SARS-CoV-2-infected patients compared to H1N1. Differential gene expression was defined as p = 0.02 and log2 fold change of 0.5.

(E) Heatmap representation of genes involved in coagulation, complement, and platelet activation and their relative expression in all SARS-CoV-2 and H1N1 infection (asterisk indicates significant genes between H1N1 and SARS-CoV-2 shown in the volcano plot). SARS-CoV-2-infected patients (n = 3), H1N1 (n = 3), and SARS-CoV-2/H1N1 (n = 1).

There was little differential gene expression in macrophages between the dual infected and H1N1 infected (Figure 4C, right panel). However, there were significant differences in macrophage gene expression in SARS-CoV-2 compared to H1N1 regions and the dual infected compared to SARS-CoV-2 (Figure 4C, left and middle panels). Gene-pathway analyses showed that, although immune signaling was observed in all groups, SARS-CoV-2 regions demonstrated enrichment of pathways related to tissue remodeling and differential activation of the innate immune signaling (Figure 4D; Figure S5B). SARS-CoV-2 macrophages displayed genes consistent with alternative macrophage activation[21] (Figure 4E).

CellPress
OPEN ACCESS

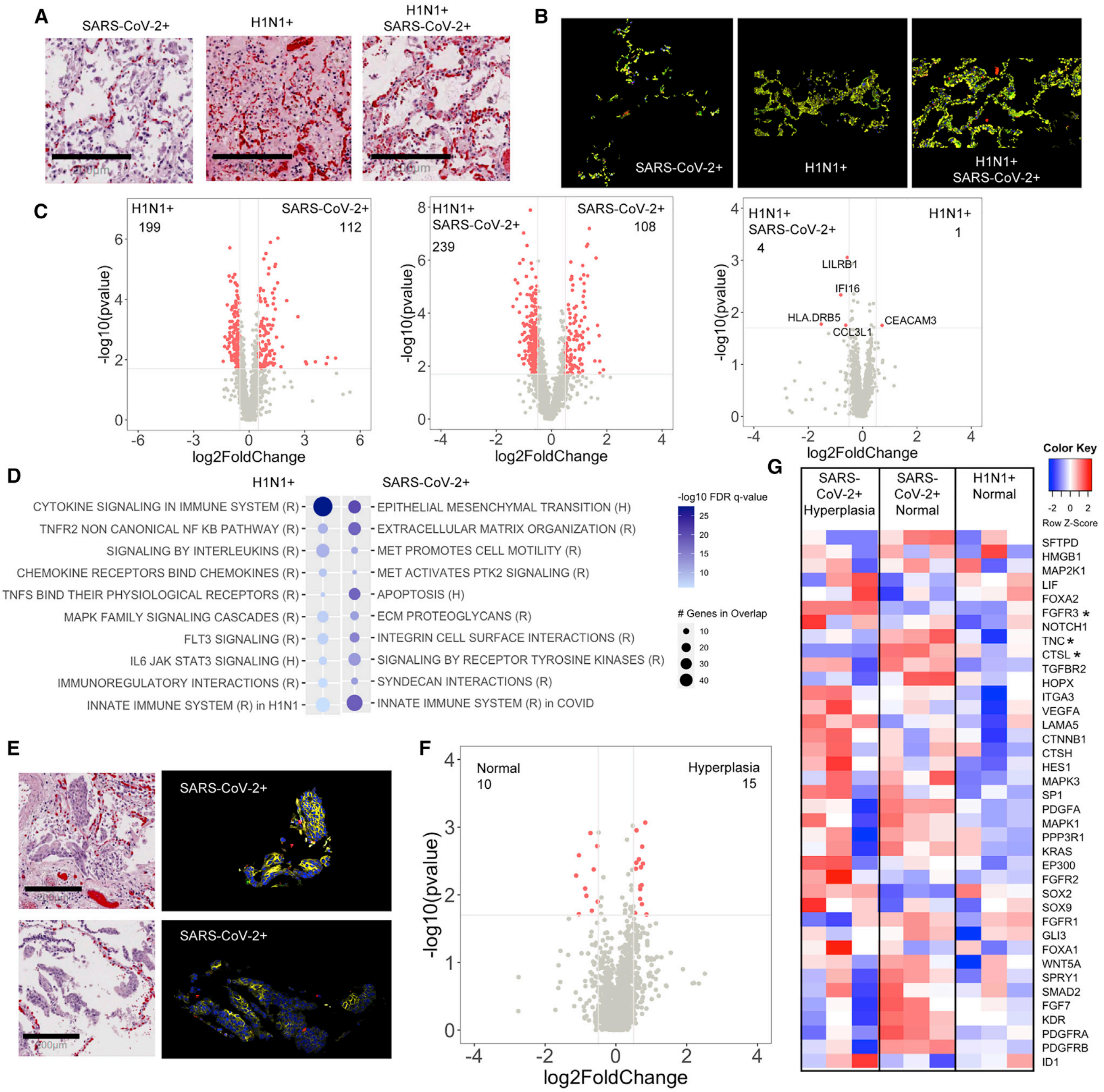

Figure 3. SARS-CoV-2 infection promotes alveolar epithelial hyperplasia

(A and B) Histological analysis of the alveolar epithelium stained (A) by H&E (scale bars, 200 μm) or (B) by immunofluorescence for EpCAM (yellow).

(C and D) Differential gene-expression analysis (C) and GSEA using reactome (R) and hallmark (H) datasets (D) for upregulated or downregulated genes in SARS-CoV-2-infected patients compared to H1N1.

(E) Histological analysis of alveolar epithelium (scale bars, 200 μm for H&E) in SARS-CoV-2 patients shows cellular hyperplasia in H&E with $EpCAM^{+}$ immunofluorescent staining (yellow).

(F) Differential gene-expression analysis of normal and hyperplastic alveolar epithelium in SARS-CoV-2-infected patients.

(G) Heatmap representation of genes involved in alveolar epithelium proliferation (GO:0060502) and their relative expression in all SARS-CoV-2 normal alveolar epithelium, hyperplastic alveolar epithelium, and H1N1 normal alveolar epithelium (asterisk indicates significant genes between H1N1 and SARS-CoV-2 shown in the volcano plot). Differential gene expression was defined as p = 0.02 and log2 fold change of 0.5. SARS-CoV-2-infected patients (n = 3), H1N1 (n = 3), and SARS-CoV-2/H1N1 (n = 1).

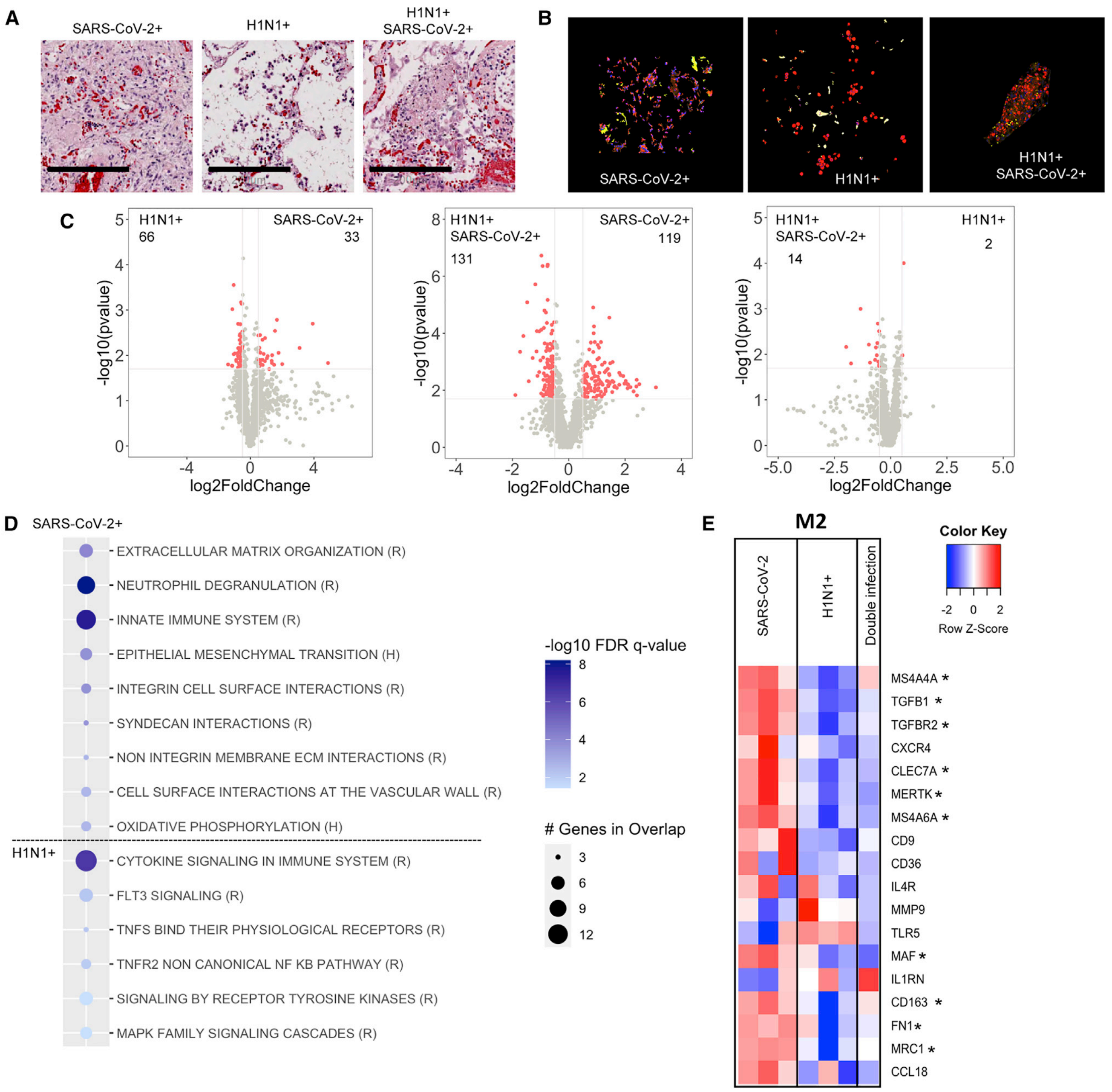

Figure 4. SARS-CoV-2 infection induces an alternative activation phenotype in lung macrophages

(A and B) Histological analysis of the lung macrophages (scale bars, 200 μm) stained by H&E (A) and immunofluorescence for CD68 (red) (B).

(C and D) Differential gene-expression analysis (C) and GSEA using reactome (R) and hallmark (H) datasets (D) for upregulated or downregulated genes in SARS-CoV-2-infected patients compared to H1N1.

(E) Heatmap representation of genes defining a pro-inflammatory (M1) or alternative activated (M2) macrophage phenotype (asterisk indicates significant genes between H1N1 and SARS-CoV-2 shown in the volcano plots). Differential gene expression was defined as p = 0.02 and log2 fold change of 0.5. SARS-CoV-2-infected patients (n = 3), H1N1 (n = 3), and SARS-CoV-2/H1N1 (n = 1).

DISCUSSION

Our study focused on examining transcriptional signatures active in viral ARDS from H1N1 and SARS-CoV-2 patients to better understand fundamental biologic processes related to these critical disease phenotypes. Although prior transcriptomic studies have focused on individual immune cells,[22,23] isolated epithelial cells,[24] or bulk lung tissue RNA,[25] these approaches do not have the benefit of maintaining tissue architecture to examine gene expression *in situ*. To date, only the study from

Desai et al.[12] addresses the heterogeneous responses in the SARS-CoV-2 lung while maintaining an intact tissue architecture. In this study, we utilized the spatial transcriptomic approach to focus on ARDS regions in SARS-CoV-2 subjects compared to other forms of virus-induced ARDS, providing the ability to identify regional or cell-specific gene regulation.

Prior studies have largely delineated the expression of altered coagulopathy as a significant gene signature.[9,11,26] Although our results validate these findings, using spatial transcriptomics, we also identify a notable enrichment in extracellular-matrix-associated genes in SARS-CoV-2 regions compared to H1N1. Prior data have shown that excessive ECM turnover portends increased mortality in other causes of ARDS.[27] Interleukin-6 (IL-6) has been associated with worse outcomes in patients with SARS-CoV-2.[28,29] In line with the study by Desai et al.,[12] we did not find major differences within the SARS-CoV-2 cohort for IL-6 expression between patients and regions of differential viral presence. It has been previously shown that IL-6 increases transforming growth factor-β (TGF-β) signaling by modulating the compartmentalization of the TGF-β receptor,[30] which is a known pro-fibrotic signaling pathway. Interestingly, in our dataset genes involved in the IL-6 signaling pathway were enriched in H1N1 patients. This differential expression may be due to diverse temporal regulation of tissue responses in these two viral infections, where early high IL-6 signaling in SARS-CoV-2 induces a quicker fibroproliferative cascade leading to more severe cases of lung injury and a higher collagen deposition observed in the terminal cases in this study.

Overall, these results challenge conventional wisdom and provide evidence for a more fibroproliferative ARDS phenotype in SARS-CoV-2 infection versus a more exudative inflammatory ARDS phenotype in H1N1 infection. This finding may explain the extended time on ventilator support often required by COVID-19 ARDS patients.[31] As we are biased with only terminal ARDS subjects, it is possible that this gene signature may represent a unique endotype for COVID-19,[32] which progresses to poorer outcomes versus other COVID-19 subjects who recover from ARDS. Our results also strongly suggest that, for these COVID-19 ARDS subjects, directed anti-fibrotic therapies may provide an important therapeutic approach to improve disease-related outcomes. Furthermore, this clear differential regulation of disease pathology warrants future studies in the temporal regulation of immune responses to airborne viruses.

The transcriptional signature from isolated cell populations were also consistent with these larger ARDS regions. ARDS-associated capillaries and arterioles from SARS-CoV-2-infected subjects show a notable upregulation of coagulopathy, complement activation, and platelet aggregation genes, highlighting that regional vessels are actively transcriptionally contributing to the development of COVID-associated vascular injury and microangiopathy.[33] Alveolar epithelial cells from SARS-CoV-2 subjects demonstrated regions of increased hyperplasia, a finding observed in multiple patients in a recent autopsy study.[8] It is interesting that both hyperplastic and non-hyperplastic SARS-CoV-2 alveolar epithelium had similar transcriptome, but both had enhanced metaplasia-related gene expression compared to H1N1 alveolar epithelia. A recent manuscript has suggested that EMT may be protective in early SARS-CoV-2 infection by reducing ACE2 expression;[34] its role in later-stage ARDS may be to induce a wound-healing response that has become dysregulated.

Macrophages have previously been shown to be central to immune response with SARS-CoV-2, and previous data suggest that they are critical to a "cytokine storm" in early SARS-CoV-2 infection.[35] Our data confirm that, in end-stage ARDS, both SARS-CoV-2 and H1N1 lungs demonstrated increased immune activation pathways, but the SARS-CoV-2 regions demonstrated alternative macrophage activation. These data, together with a lack of differences in gene expression observed between ARDS regions characterized with either increased or low viral staining, suggest that the patients included in this study may be part of the low-viral-load group described in the study by Desai et al.[12] In that study, patients with higher viral loads presented a macrophage phenotype skewed toward an M1-like activation paired with increased interferon responses. Furthermore, they suggest that a broad, tissue-based transcriptome response in this patient group may not impacted by viral presence during end-stage ARDS in both SARS-CoV-2- and H1N1-infected patients.

Overall, these results provide further evidence of a more fibroproliferative response to the SARS-CoV-2 versus H1N1. Future studies should also work to define the transcriptome and activity of both innate[36] and adaptive[37,38] immunity in the lungs of patients with persistent viral infection and injury and their relative contributions to the SARS-CoV-2 ARDS phenotype. Furthermore, future studies should also address the mechanisms leading to the discrepant transcriptional response observed in H1N1 and SARS-CoV-2 ARDS, as the autopsy specimens used in this study limit the ability to address the kinetics of differential ARDS development.

We also identified a co-infected individual who, upon virus staining, demonstrated a much higher burden of H1N1 compared to SARS-CoV-2 throughout the lungs. This may underscore why this individual's transcriptome more closely related to the H1N1 subjects. It is important to note that this subject had known lung disease with CT-ILD and COPD and was treated with baseline methotrexate and steroids prior to hospitalization, which may have made this individual more susceptible to dual viral infection. A small clinical series of co-infections with SARS-CoV-2 and influenza has been reported,[39] and, in these three cases, there was no obvious evidence of baseline immunosuppression noted. Although the current influenza season has seen reduced cases of hospitalized patients, it is imperative to consider that if both viruses are highly prevalent in the future, co-infection in both immunosuppressed and immunocompetent individuals may be a more frequent occurrence.

This method of analysis has uncovered pathways that augment tissue injury in SARS-CoV-2 individuals, and future studies should examine additional critically ill subjects and focus on further defining these critical pathways. It is our hope that these pathways will improve our understanding of mechanisms leading to progressive worsening of gas exchange and increased mortality in SARS-CoV-2-related ARDS. As a result, the potential for new therapeutic targets to alter the fibroproliferative response will present the potential to improve clinical outcomes in patients with progressive lung injury.

Limitations of study

The numbers of subjects included in this study are relatively limited, even though the SARS-CoV-2 and H1N1 subjects were relatively well matched for demographic features, severity of disease, and co-morbidities. These features strongly suggest that the transcriptional changes observed were due to the viral lung injury, although future studies with additional subjects would certainly be warranted. Furthermore, this study focused only on the lung parenchyma, and future studies addressing spatial heterogeneity (larger and smaller airways, lung parenchyma versus lumen) are needed. Finally, patient samples used in this study represent a discrete patient population, where disease was most severe. Future studies addressing the temporal regulation of disease will help better understand the differences observed in these two viral infections.

STAR★METHODS

Detailed methods are provided in the online version of this paper and include the following:

- KEY RESOURCES TABLE
- RESOURCE AVAILABILITY
  - Lead contact
  - Materials availability
  - Data and code availability
- EXPERIMENTAL MODEL AND SUBJECT DETAILS
  - Human subjects
- METHOD DETAILS
  - Histology
  - Immunofluorescence
  - GeoMX digital spatial profiling
- QUANTIFICATION AND STATISTICAL ANALYSIS

SUPPLEMENTAL INFORMATION

Supplemental information can be found online at https://doi.org/10.1016/j.xcrm.2021.100242.

ACKNOWLEDGMENTS

We thank the Tissue Biorepository Core Facility at UAB for their assistance with the processing of the histological specimens, as well Dr. Dezhi Wang at the UAB Pathology Core Research Laboratory, Dr. Robert Grabski at the UAB High Resolution Imaging Facility, and Dr. Peter Anderson (DVM, PhD) for their help with the immunofluorescence staining protocol and image acquisition. We also thank Erik Marchese at Nanostring for insight into the GeoMX platform. Finally, we thank Drs. Anuj Gaggar, Derek Russell, J. Michael Wells, Gregory Payne, Bryan Garcia, Stefanie Krick, and J. Edwin Blalock for their insightful feedback regarding the paper. This study was supported by the National Institutes of Health: NIGMS (K12 GM088010 to M.C.M.), NHLBI (HL102371 to A.G.; HL153113 to R.P.P. and A.G.; HL105346 to A.G. and S.W.R.); Cystic Fibrosis Foundation: Research Development Program Grant ROWE19R0 to C.M.; and Veterans Administration: VA Merit Review 1I01CX001969 to A.G.

AUTHOR CONTRIBUTIONS

C.M. and A.G. conceived the study and wrote the manuscript; P.B. performed the autopsy and oversaw the selection of pathological areas of interest; C.M. and M.C.M. performed the immunofluorescence staining with help from N.A.; N.S.S., S.W.R., and A.G. curated the clinical data; A.G. and R.P.P. obtained IRB approval for the study and extensively edited the manuscript; L.Z., K.T., and Y.L. performed the Nanostring RNA experiment and initial data analysis.

DECLARATION OF INTERESTS

K.T., Y.L., and L.Z. are employees of Nanostring technologies. There is no financial conflict of interest to disclose for this study (all services provided by Nanostring technologies were paid under the grants awarded to the senior author).

Received: August 6, 2020
Revised: November 16, 2020
Accepted: March 16, 2021
Published: March 23, 2021

REFERENCES

1. Narula, T., Safley, M., and deBoisblanc, B.P. (2010). H1N1-associated acute respiratory distress syndrome. Am. J. Med. Sci. *340*, 499–504.
2. Wu, F., Zhao, S., Yu, B., Chen, Y.M., Wang, W., Song, Z.G., Hu, Y., Tao, Z.W., Tian, J.H., Pei, Y.Y., et al. (2020). A new coronavirus associated with human respiratory disease in China. Nature *579*, 265–269.
3. Wang, D., Hu, B., Hu, C., Zhu, F., Liu, X., Zhang, J., Wang, B., Xiang, H., Cheng, Z., Xiong, Y., et al. (2020). Clinical Characteristics of 138 Hospitalized Patients With 2019 Novel Coronavirus-Infected Pneumonia in Wuhan China. JAMA *323*, 1061–1069.
4. Auld, S.C., Caridi-Scheible, M., Blum, J.M., Robichaux, C., Kraft, C., Jacob, J.T., Jabaley, C.S., Carpenter, D., Kaplow, R., Hernandez-Romieu, A.C., et al.; Emory COVID-19 Quality and Clinical Research Collaborative (2020). ICU and Ventilator Mortality Among Critically Ill Adults With Coronavirus Disease 2019. Crit. Care Med. *48*, e799–e804.
5. Bhatraju, P.K., Ghassemieh, B.J., Nichols, M., Kim, R., Jerome, K.R., Nalla, A.K., Greninger, A.L., Pipavath, S., Wurfel, M.M., Evans, L., et al. (2020). Covid-19 in Critically Ill Patients in the Seattle Region - Case Series. N. Engl. J. Med. *382*, 2012–2022.
6. Jain, S., Kamimoto, L., Bramley, A.M., Schmitz, A.M., Benoit, S.R., Louie, J., Sugerman, D.E., Druckenmiller, J.K., Ritger, K.A., Chugh, R., et al.; 2009 Pandemic Influenza A (H1N1) Virus Hospitalizations Investigation Team (2009). Hospitalized patients with 2009 H1N1 influenza in the United States, April-June 2009. N. Engl. J. Med. *361*, 1935–1944.
7. Auvinen, R., Nohynek, H., Syrjänen, R., Ollgren, J., Kerttula, T., Mäntylä, J., Ikonen, N., Loginov, R., Haveri, A., Kurkela, S., et al. (2020). Comparison of the clinical characteristics and outcomes of hospitalized adult COVID-19 and influenza patients: a prospective observational study. medRxiv, 2020.06.29.20140632.
8. Carsana, L., Sonzogni, A., Nasr, A., Rossi, R.S., Pellegrinelli, A., Zerbi, P., Rech, R., Colombo, R., Antinori, S., Corbellino, M., et al. (2020). Pulmonary post-mortem findings in a series of COVID-19 cases from northern Italy: a two-centre descriptive study. Lancet Infect. Dis. *20*, 1135–1140.
9. Goshua, G., Pine, A.B., Meizlish, M.L., Chang, C.H., Zhang, H., Bahel, P., Baluha, A., Bar, N., Bona, R.D., Burns, A.J., et al. (2020). Endotheliopathy in COVID-19-associated coagulopathy: evidence from a single-centre, cross-sectional study. Lancet Haematol. *7*, e575–e582.
10. Xu, J., Xu, X., Jiang, L., Dua, K., Hansbro, P.M., and Liu, G. (2020). SARS-CoV-2 induces transcriptional signatures in human lung epithelial cells that promote lung fibrosis. Respir. Res. *21*, 182.
11. Ackermann, M., Verleden, S.E., Kuehnel, M., Haverich, A., Welte, T., Laenger, F., Vanstapel, A., Werlein, C., Stark, H., Tzankov, A., et al. (2020). Pulmonary Vascular Endothelialitis, Thrombosis, and Angiogenesis in Covid-19. N. Engl. J. Med. *383*, 120–128.
12. Desai, N., Neyaz, A., Szabolcs, A., Shih, A.R., Chen, J.H., Thapar, V., Nieman, L.T., Solovyov, A., Mehta, A., Lieb, D.J., et al. (2020). Temporal and Spatial Heterogeneity of Host Response to SARS-CoV-2 Pulmonary Infection. medRxiv. https://doi.org/10.1101/2020.07.30.20165241.

13. Lai, C.C., Wang, C.Y., and Hsueh, P.R. (2020). Co-infections among patients with COVID-19: The need for combination therapy with non-anti-SARS-CoV-2 agents? J. Microbiol. Immunol. Infect. *53*, 505–512.
14. Ware, L.B., and Matthay, M.A. (2000). The acute respiratory distress syndrome. N. Engl. J. Med. *342*, 1334–1349.
15. Rieg, A.D., Suleiman, S., Anker, C., Verjans, E., Rossaint, R., Uhlig, S., and Martin, C. (2018). PDGF-BB regulates the pulmonary vascular tone: impact of prostaglandins, calcium, MAPK- and PI3K/AKT/mTOR signalling and actin polymerisation in pulmonary veins of guinea pigs. Respir. Res. *19*, 120.
16. Marconett, C.N., Zhou, B., Sunohara, M., Pouldar, T.M., Wang, H., Liu, Y., Rieger, M.E., Tran, E., Flodby, P., Siegmund, K.D., et al. (2017). Cross-Species Transcriptome Profiling Identifies New Alveolar Epithelial Type I Cell-Specific Genes. Am. J. Respir. Cell Mol. Biol. *56*, 310–321.
17. Greaves, D.R., and Gordon, S. (2002). Macrophage-specific gene expression: current paradigms and future challenges. Int. J. Hematol. *76*, 6–15.
18. Whyte, C.S., Morrow, G.B., Mitchell, J.L., Chowdary, P., and Mutch, N.J. (2020). Fibrinolytic abnormalities in acute respiratory distress syndrome (ARDS) and versatility of thrombolytic drugs to treat COVID-19. J. Thromb. Haemost. *18*, 1548–1555.
19. Budinger, G.R., and Sznajder, J.I. (2006). The alveolar-epithelial barrier: a target for potential therapy. Clin. Chest Med. *27*, 655–669, abstract ix.
20. Snelgrove, R.J., Godlee, A., and Hussell, T. (2011). Airway immune homeostasis and implications for influenza-induced inflammation. Trends Immunol. *32*, 328–334.
21. Orecchioni, M., Ghosheh, Y., Pramod, A.B., and Ley, K. (2019). Macrophage Polarization: Different Gene Signatures in M1(LPS+) vs. Classically and M2(LPS-) vs. Alternatively Activated Macrophages. Front. Immunol. *10*, 1084.
22. Liao, M., Liu, Y., Yuan, J., Wen, Y., Xu, G., Zhao, J., Cheng, L., Li, J., Wang, X., Wang, F., et al. (2020). Single-cell landscape of bronchoalveolar immune cells in patients with COVID-19. Nat. Med. *26*, 842–844.
23. Wilk, A.J., Rustagi, A., Zhao, N.Q., Roque, J., Martínez-Colón, G.J., McKechnie, J.L., Ivison, G.T., Ranganath, T., Vergara, R., Hollis, T., et al. (2020). A single-cell atlas of the peripheral immune response in patients with severe COVID-19. Nat. Med. *26*, 1070–1076.
24. Cai, G., Bossé, Y., Xiao, F., Kheradmand, F., and Amos, C.I. (2020). Tobacco Smoking Increases the Lung Gene Expression of ACE2, the Receptor of SARS-CoV-2. Am. J. Respir. Crit. Care Med. *201*, 1557–1559.
25. Pinto, B.G.G., Oliveira, A.E.R., Singh, Y., Jimenez, L., Gonçalves, A.N.A., Ogava, R.L.T., Creighton, R., Schatzmann Peron, J.P., and Nakaya, H.I. (2020). ACE2 Expression Is Increased in the Lungs of Patients With Comorbidities Associated With Severe COVID-19. J. Infect. Dis. *222*, 556–563.
26. Magro, C., Mulvey, J.J., Berlin, D., Nuovo, G., Salvatore, S., Harp, J., Baxter-Stoltzfus, A., and Laurence, J. (2020). Complement associated microvascular injury and thrombosis in the pathogenesis of severe COVID-19 infection: A report of five cases. Transl. Res. *220*, 1–13.
27. Clark, J.G., Milberg, J.A., Steinberg, K.P., and Hudson, L.D. (1995). Type III procollagen peptide in the adult respiratory distress syndrome. Association of increased peptide levels in bronchoalveolar lavage fluid with increased risk for death. Ann. Intern. Med. *122*, 17–23.
28. Grifoni, E., Valoriani, A., Cei, F., Lamanna, R., Gelli, A.M.G., Ciambotti, B., Vannucchi, V., Moroni, F., Pelagatti, L., Tarquini, R., et al. (2020). Interleukin-6 as prognosticator in patients with COVID-19. J. Infect. *81*, 452–482.
29. Herold, T., Jurinovic, V., Arnreich, C., Lipworth, B.J., Hellmuth, J.C., von Bergwelt-Baildon, M., Klein, M., and Weinberger, T. (2020). Elevated levels of IL-6 and CRP predict the need for mechanical ventilation in COVID-19. J. Allergy Clin. Immunol. *146*, 128–136.
30. Zhang, X.L., Topley, N., Ito, T., and Phillips, A. (2005). Interleukin-6 regulation of transforming growth factor (TGF)-beta receptor compartmentalization and turnover enhances TGF-beta1 signaling. J. Biol. Chem. *280*, 12239–12245.
31. Zangrillo, A., Beretta, L., Scandroglio, A.M., Monti, G., Fominskiy, E., Colombo, S., Morselli, F., Belletti, A., Silvani, P., Crivellari, M., et al. (2020). Characteristics, treatment, outcomes and cause of death of invasively ventilated patients with COVID-19 ARDS in Milan, Italy. Crit. Care Resusc., Published online April 23, 2020.
32. Hariri, L., and Hardin, C.C. (2020). Covid-19, Angiogenesis, and ARDS Endotypes. N. Engl. J. Med. *383*, 182–183.
33. Becker, R.C. (2020). COVID-19 update: Covid-19-associated coagulopathy. J. Thromb. Thrombolysis *50*, 54–67.
34. Stewart, C.A., Gay, C.M., Ramkumar, K., Cargill, K.R., Cardnell, R.J., Nilsson, M.B., Heeke, S., Park, E.M., Kundu, S.T., Diao, L., et al. (2020). SARS-CoV-2 infection induces EMT-like molecular changes, including ZEB1-mediated repression of the viral receptor ACE2, in lung cancer models. bioRxiv, Published online May 29, 2020. https://doi.org/10.1101/2020.05.28.122291.
35. Merad, M., and Martin, J.C. (2020). Pathological inflammation in patients with COVID-19: a key role for monocytes and macrophages. Nat. Rev. Immunol. *20*, 355–362.
36. Gasteiger, G., D'Osualdo, A., Schubert, D.A., Weber, A., Bruscia, E.M., and Hartl, D. (2017). Cellular Innate Immunity: An Old Game with New Players. J. Innate Immun. *9*, 111–125.
37. Chiu, C., Ellebedy, A.H., Wrammert, J., and Ahmed, R. (2015). B cell responses to influenza infection and vaccination. Curr. Top. Microbiol. Immunol. *386*, 381–398.
38. Blank, C.U., Haining, W.N., Held, W., Hogan, P.G., Kallies, A., Lugli, E., Lynn, R.C., Philip, M., Rao, A., Restifo, N.P., et al. (2019). Defining 'T cell exhaustion'. Nat. Rev. Immunol. *19*, 665–674.
39. Konala, V.M., Adapa, S., Naramala, S., Chenna, A., Lamichhane, S., Garlapati, P.R., Balla, M., and Gayam, V. (2020). A Case Series of Patients Coinfected With Influenza and COVID-19. J. Investig. Med. High Impact Case Rep. *8*, Published online June 10, 2020. https://doi.org/10.1177/2324709620934674.
40. Johnson, W.E., Li, C., and Rabinovic, A. (2007). Adjusting batch effects in microarray expression data using empirical Bayes methods. Biostatistics *8*, 118–127.
41. Leek, J.T., Johnson, W.E., Parker, H.S., Jaffe, A.E., and Storey, J.D. (2012). The sva package for removing batch effects and other unwanted variation in high-throughput experiments. Bioinformatics *28*, 882–883.

STAR★METHODS

KEY RESOURCES TABLE

REAGENT or RESOURCE	SOURCE	IDENTIFIER
**Antibodies**		
SARS-CoV-2 nucleocapsid	GeneTex	Cat#: GTX135361 RRID: AB_2887484
H1N1	Abcam	Cat#ab20343, RRID:AB_445525
CD68	Abcam	Cat# ab224029, clone:EPR20545
EpCAM	Abcam	Cat# ab213500, clone:EPR220532-222
alpha-SMA	Invitrogen	Cat# 53-9760-82, RRID:AB_2574461
KRT19	Thermofisher	Cat# TA803863, clone: OTI6A8
TGFBR2	Thermofisher	Cat# TA807912, clone: OTI3B4
F13A1	Abcam	Cat# ab225018, clone: EP3372
CD68	Santa Cruz	Cat# sc-20060 AF647, RRID:AB_627158
**Biological samples**		
Patient paraffine embedded lung tissue	UAB Tissue Biorepository Core Facility	https://sites.uab.edu/tissuebank/
**Chemicals, peptides, and recombinant proteins**		
Xylene	Fisher Scientific	Cat#X5P-1GAL
100% Ethanol	Fisher Scientific	Cat#HC-800-1GL
95% Ethanol	Fisher Scientific	Cat#HC-1100-1GL
Tris-EDTA ph9 buffer	Abcam	Cat#ab93684
Bovine Serum Albumin Fraction V	Fisher Scientific	Cat#BP1605-100
R-Phycoerythrin lightning-link labeling kit	Novus Biologicals	Cat#703-0010
Alexa 647 labeling kit	Thermofisher	Cat# A20186
PHEM buffer	Goldbio	https://www.goldbio.com/documents/3553/PHEM+Buffer+4X+Stock+Solution.pdf
DAPI	Biolegend	Cat#422801
ProLong Gold antifade mounting media	Thermofisher	Cat#P36934
**Critical commercial assays**		
GeoMx Cancer Transcriptome Atlas (COVID-19)	Nanostring	https://www.nanostring.com/products/geomx-digital-spatial-profiler/geomx-rna-assays/geomx-cancer-transcriptome-atlas/
**Deposited data**		
Transcriptomics data	This paper	https://doi.org/10.17632/n5dn4xzg7j.1
**Software and algorithms**		
Prism v7	GraphPad	https://www.graphpad.com/scientific-software/prism/
R v3.5.2	R project	https://www.r-project.org/
Microsoft Excel	Microsoft	https://www.microsoft.com/en-us/microsoft-365/p/excel/cfq7ttc0k7dx?activetab=pivot:overviewtab
Adobe Illustrator 2020	Adobe	https://www.adobe.com/products/illustrator.html
GSEA	Broad Insititute	https://www.gsea-msigdb.org/gsea/index.jsp
GeoMx DSP data center	Nanostring	https://www.nanostring.com/products/geomx-digital-spatial-profiler/geomx-data-center/

RESOURCE AVAILABILITY

Lead contact

Further information and requests for resources and reagents should be directed to and will be fulfilled by the Lead Contact, Dr. Amit Gaggar (agaggar@uabmc.edu).

Materials availability

This study did not generate new unique reagents.

Data and code availability

The datasets generated during this study are available on Mendeley: https://doi.org/10.17632/n5dn4xzg7j.1.

EXPERIMENTAL MODEL AND SUBJECT DETAILS

Human subjects

Pulmonary autopsy specimens were collected from patients deceased due to ARDS. Three patients were infected with SARS-CoV-2, three patients were infected with influenza A subtype H1N1, and one patient was infected with both SARS-CoV-2 and H1N1. The study was approved by the Institutional Review Board (UAB-IRB 300005258, VA-IRB 1573682) and summary demographic and clinical data are presented in Table S1.

METHOD DETAILS

Histology

Lungs were inflated isobarically with 10% formalin and preserved in paraffin blocks. Sequential tissue sections of 5μm were used for viral staining by immunofluorescence, for RNA analysis, and for hematoxylin and eosin or Masson's trichrome staining to identify ARDS pathological features and collagen deposition respectively.

Immunofluorescence

Paraffin-embedded tissue slides were incubated for 2 hours at 60°C. Deparaffinization and rehydration of the slides was performed with sequential three 5 minutes incubations in xylene (Fisher Scientific), two sequential 5 minutes incubations in 100% denatured ethyl alcohol (Fisher Scientific), two sequential 5 minutes incubations in 95% denatured ethyl alcohol (Fisher Scientific), followed by three sequential 5 minutes incubations in distilled water under 40rpm gentle shaking. Antigen retrieval was performed in pre-warmed Tris-EDTA pH 9 buffer (Abcam) at 70°C in heated steamer for 20 minutes, followed by three washes in distilled water under 40rpm gentle shaking. Tissue sections were blot dry and incubated with PBS for 10 minutes at room temperature, followed by blockade of non-specific binding using 3% w/v BSA (Fisher Scientific) for 40 minutes at room temperature. The SARS-CoV-2 only infected slides and the SARS-CoV-2/H1N1 slide were stained for 1 hour at room temperature with anti-SARS-CoV-2 nucleocapsid antibody (GeneTex, GTX135361, RRID: AB_2887484) directly labeled with R-Phycoerythrin lightning-link labeling kit (Novus Biologicals) at 1:500 dilution in PBS, 3% w/v BSA. Slides were then washed three times for 5 minutes in PBS under gentle agitation.

The SARS-CoV-2/H1N1 slide and the H1N1 only infected slides were then fixed for 10 minutes at room temperature with 4% paraformaldehyde and washed three times with PBS, 0.1% Tween-20 (PBST) for 5 minutes. Staining for H1N1 was performed with Alexa 647 (Thermofisher) pre-labeled anti-influenza A virus nucleoprotein antibody (Abcam, ab20343, RRID:AB_445525) at 1:200 in PHEM buffer (Goldbio) for 1 hour at room temperature. Tissues were then washed three times in PBST for 5 minutes.

Staining for EpCAM (Abcam, ab213500, clone: EPR20532-222, 1:200 in 3% BSA), CD68 (Abcam, ab224029, clone: EPR20545, 1:1000 in PHEM buffer), alpha-smooth muscle actin (Invitrogen, 53-9760-82, clone:1A4, RRID: AB_2574461 1:400 in PHEM buffer), KRT19 (Thermofisher, TA803863, clone:OTI6A8, 1: 1:500 in 3% BSA), TGFBR (Thermofisher, TA807912, clone:OTI3B4, 1:500 in PHEM buffer), and F13A1 (Abcam, ab225018, clone:EP3372, 1:500 in PHEM buffer) was performed for 1 hour at room temperature and then washed three times for 5 minutes in PBST under gentle agitation.

Nuclei counter staining for all slides was performed using 300nM DAPI (Biolegend) in PBS for 5 minutes, followed by three 5 minutes washes in PBS. Slides were mounted using ProLong Gold antifade mounting media with DAPI (Thermofisher) and stored in the dark until image acquisition. Confocal immunofluorescence images were acquired using the Nikon A1R confocal microscope.

GeoMX digital spatial profiling

Paraffin embedded tissues were processed and analyzed at NanoString techonology laboratories using a combination of fluorescently labeled antibodies, anti-CD68 (Santa Cruz, sc-20060 AF647, clone: KP1, RRID: AB_627158), anti-EpCAM (Abcam, ab213500, clone:EPR20532-222), anti-smooth muscle actin (Invitrogen, 53-9760-82, clone:1A4, RRID: AB_2574461) and the GeoMX COVID-19 Immune Response Atlas gene set with custom probe set specific for SARS-CoV-2 lung infection and tissue responses (see Table S2 for SARS-CoV-2 related gene list and Figure S1 for workflow), totaling 1860 genes. Selection of regions of interests (ROI, 12 per patient) was performed based on the immunofluorescent viral staining, the cellular immunofluorescent profile and the pathological features of ARDS (i.e., presence of hyaline membranes and diffused alveolar damage) observed in the H&E stained sections. To ensure even and representative selection of ROIs, lower left lobe of the lung was analyzed in all subjects. Each patient had 2-4 total lung areas selected in regions of ARDS (confirmed by pathologist), 2-4 ROIs for epithelial cells (normal epithelium versus hyperplastic), 2-3 vascular beds selected, and 2-4 macrophage populations (infiltrate and clusters). For cell-specific profiling (Figures 2, 3, and 4), at least 50 cells per ROI were utilized for analyses.

QUANTIFICATION AND STATISTICAL ANALYSIS

Sequencing through the Nanostring GeoMx platform is performed on the RNA probe tag and not on the transcript itself, providing less sequencing bias and a more accurate transcript count. RNA probe counts used in the analyses were selected following a sequencing QC according to Nanostring protocols, where counts from each area of interest are analyzed and under-sequenced samples are dropped (field of view percentage of 75% and Binding density from 0.1 to 2.25), and a probe QC, where mRNAs are targeted by multiple probes and outlier probes are dropped from downstream data analysis (positive spike-in normalization factor between 0.3 and 3). Then RNA counts were normalized using a signal-based normalization, in which individual counts are normalized against the 75th percentile of signal from their own area of interest. The final list of detectable genes was then obtained by dropping genes in each specific group (ARDS regions, vascular, epithelium, macrophages) by using a limit of quantification (LOQ) of 20% coverage within replicates. The LOQ was calculated using the geometric mean and geometric standard deviation of negative probes in the dataset. Counts were normalized to log2 and statistical comparisons were performed using a two sample t test upon normality testing and ComBat correction for batch effect[40,41]. Comparison of SARS-CoV-2 to H1N1 was performed by averaging the technical replicates and by comparing biological replicates (n = 3 per group). Comparison of the double infected patient to the single infection was done using technical replicates (ROIs) as unique samples for statistical reasons. P value threshold for differential gene expression were set at p = 0.02 and log2 fold change of 0.5. All details for the statistical analyses and number of replicates can be found in the figure legends. All analyses for the volcano plots can be found in the Data S1 file.

Statistical analysis done for specific gene expression (Figure S4) was performed using Mann-Whitney test and data are shown as median and interquartile range (n = 3 per group).

Cell Reports Medicine, Volume *2*

Supplemental information

Spatial mapping of SARS-CoV-2 and H1N1 lung injury identifies differential transcriptional signatures

Camilla Margaroli, Paul Benson, Nirmal S. Sharma, Matthew C. Madison, Sarah W. Robison, Nitin Arora, Kathy Ton, Yan Liang, Liang Zhang, Rakesh P. Patel, and Amit Gaggar

Supplementary Materials for

Spatial mapping of SARS-CoV-2 and H1N1 Influenza Lung Injury Identifies Differential Transcriptional Signatures

C. Margaroli, P. Benson, N.S Sharma, M.C. Madison, S.W. Robison, N. Arora, K. Ton, Y. Liang, L. Zhang, R.P. Patel, A. Gaggar

Correspondence to: agaggar@uabmc.edu

This PDF file includes:

Figs. S1 to S6
Tables S1 to S2

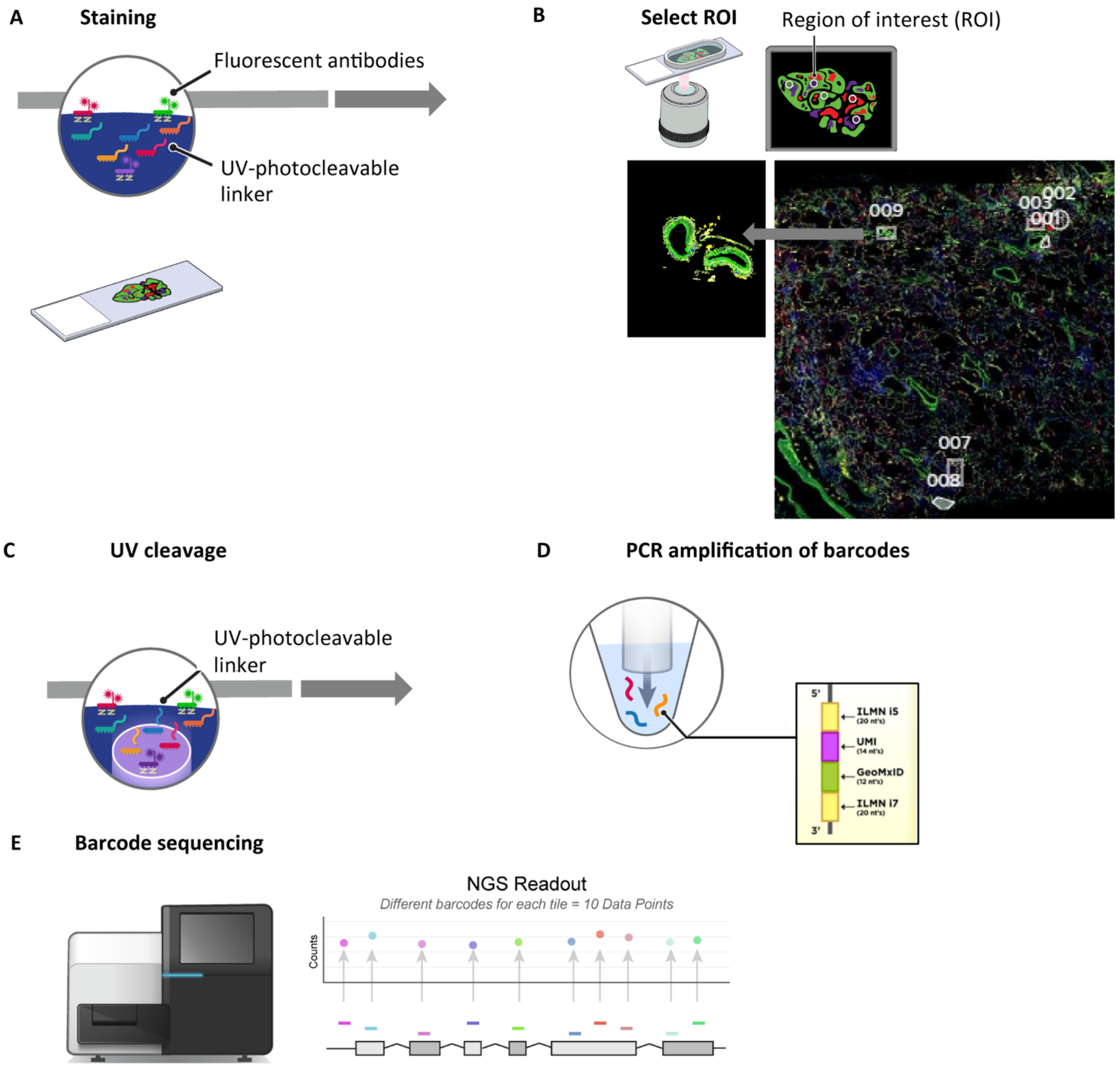

Fig. S1 related to STAR Methods. Nanostring workflow. (A) The tissue is processed and stained using a standard ISH protocol with both fluorescent labeled morphology markers and sequence specific RNA probes. **(B)** Morphology markers are used for Region of Interest (ROI) and Area of Interest (AOI) selection. **(C)** After ROI/AOI selection, UV light is shone on the ROI/AOI decoupling the barcodes from the RNA probes only from the selected area or cells of interest. Five probes per mRNA target are used and are coupled to molecular barcodes and a unique barcode identifies a specific gene target. The UMI identifies a specific molecule and accounts for amplification bias from PCR. **(D)** RNA probes for the ROI/AOI are aspirated and deposited into a unique well of a 96 well plate. The PCR step adds dual indexing barcodes (Index1 and Index2) to identify AOIs and adds Illumina flow cell adapter regions (P5 and P7) to enable sequencing. **(E)** The barcodes are sequenced.

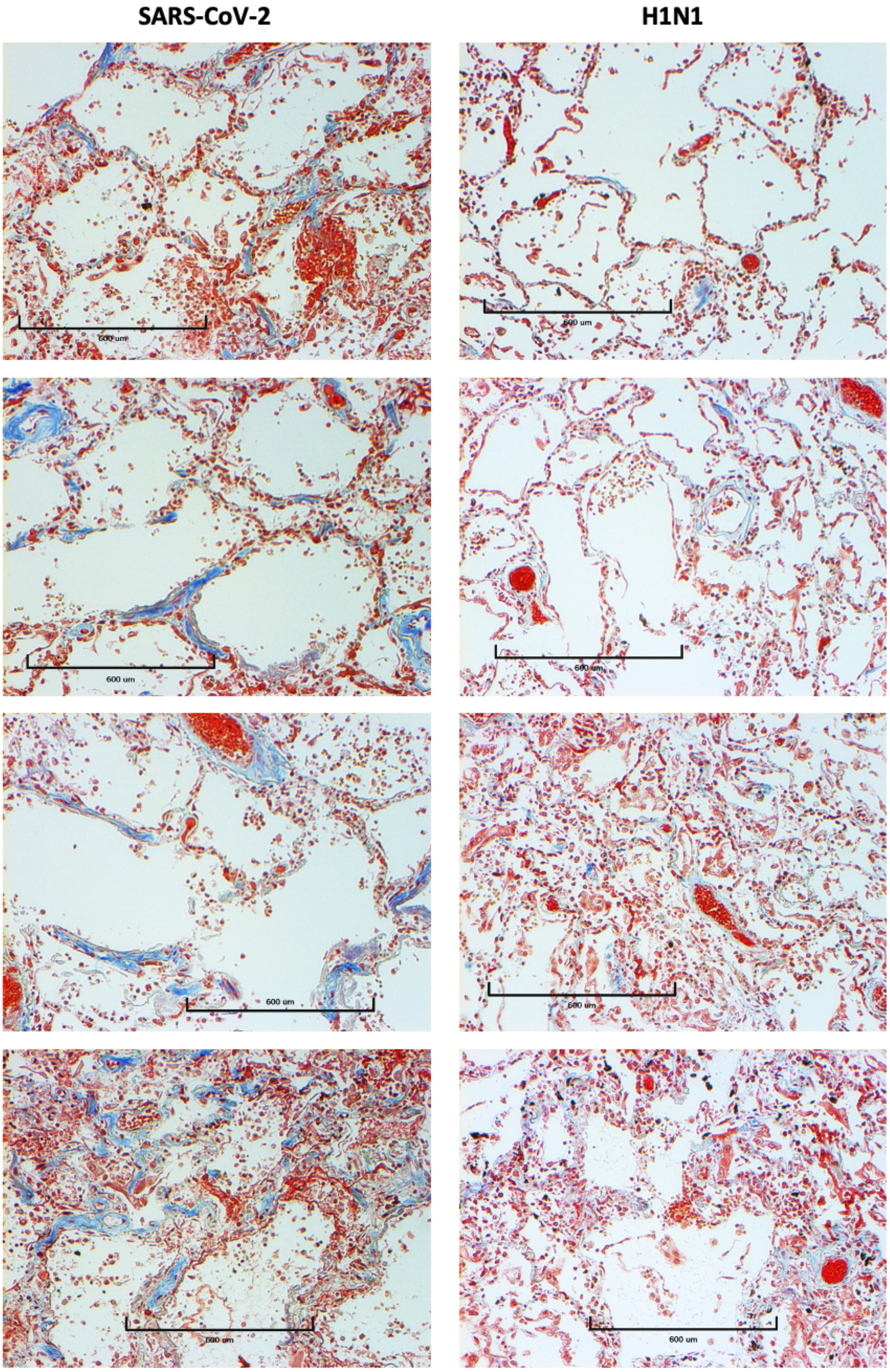

Fig. S2 related to Figure 1. Representative areas of collagen deposition in H1N1 and SARS-CoV-2 lung tissue. Collagen staining (blue) was performed by Masson's trichrome staining on paraffin embedded lung tissues. Scale bar 600μm. SARS-CoV-2 infected patients (n=3), H1N1 (n=3).

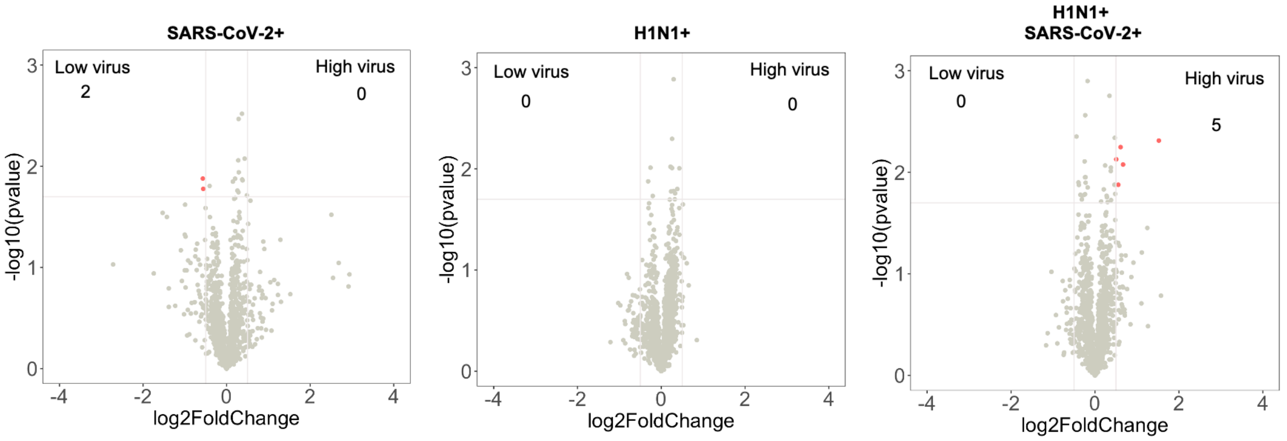

Fig. S3 related to Figure 1. Viral load does not influence gene expression. Differential gene expression comparing areas of high and low viral presence determined by immunofluorescence in patients infected by SARS-CoV-2, H1N1 or both viruses, showed no differential modulation of gene expression. P-value threshold for differential gene expression were set at p=0.02 and log2 fold change of 0.5. Biological replicates were analyzed for SARS-CoV-2 infected patients (n=3) and H1N1 (n=3), technical replicates were used for SARS-CoV-2/H1N1 (high n=2, low n=2).

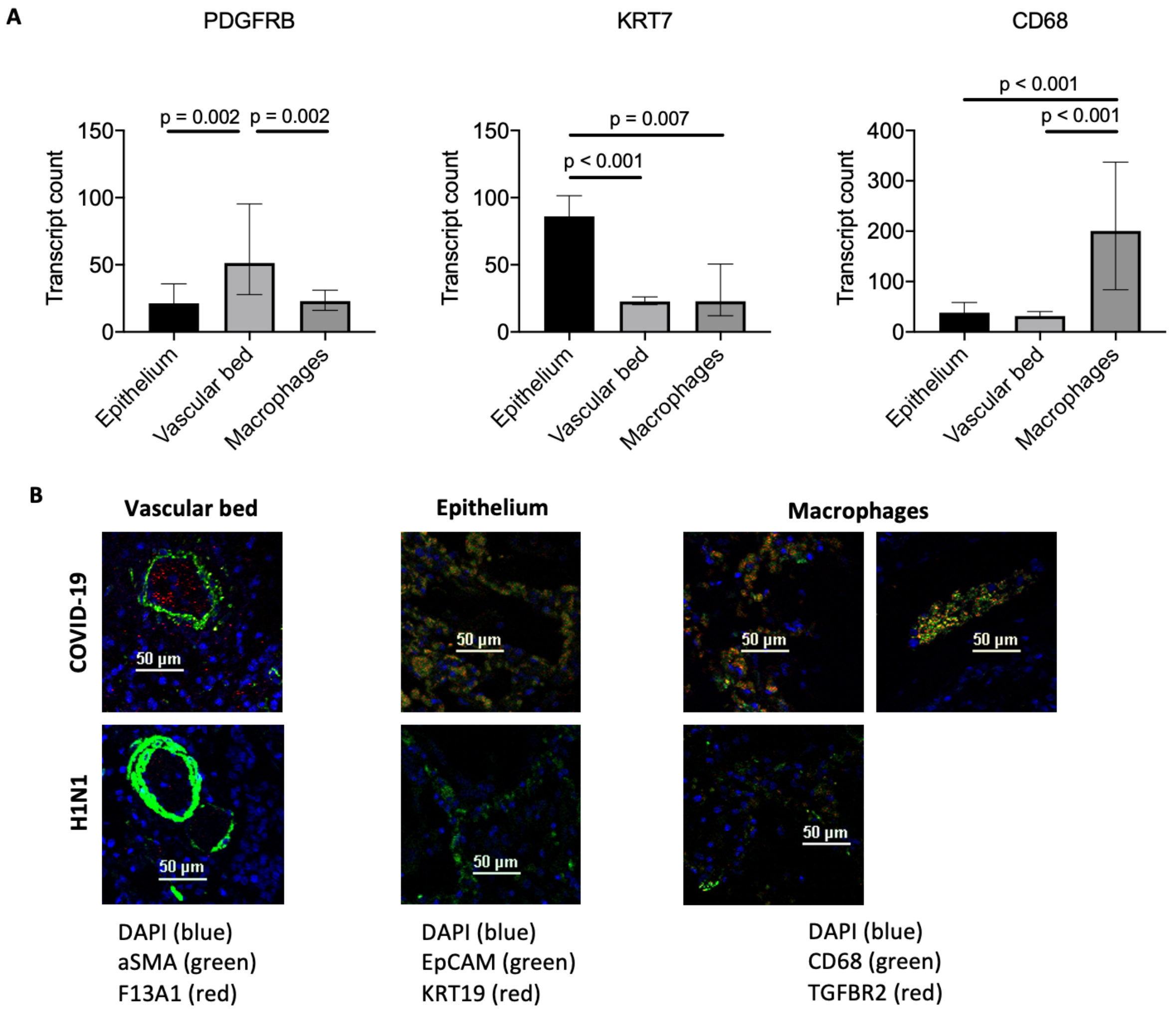

Fig. S4 related to Figures 2, 3, and 4. Gene and protein expression in cell populations selected by immunofluorescence. Specific cell populations for transcriptional profiling were selected using immunofluorescence staining. **(A)** Validation of cellular purity was performed by gene expression in the selected samples, for vascular bed (PDGFRB), alveolar epithelium (KRT7), and macrophages (CD68). Replicates for expression include SARS-CoV-2 infected patients (n=3), H1N1 (n=3), and SARS-CoV-2/H1N1 (n=1). **(B)** Validation of colocalization for high expressed genes in SARS-CoV-2 tissue for epithelium, vascular bed and macrophages. Scale bars= 50μm. Results are shown as median and interquartile range. Data were analyzed using Mann-Whitney test.

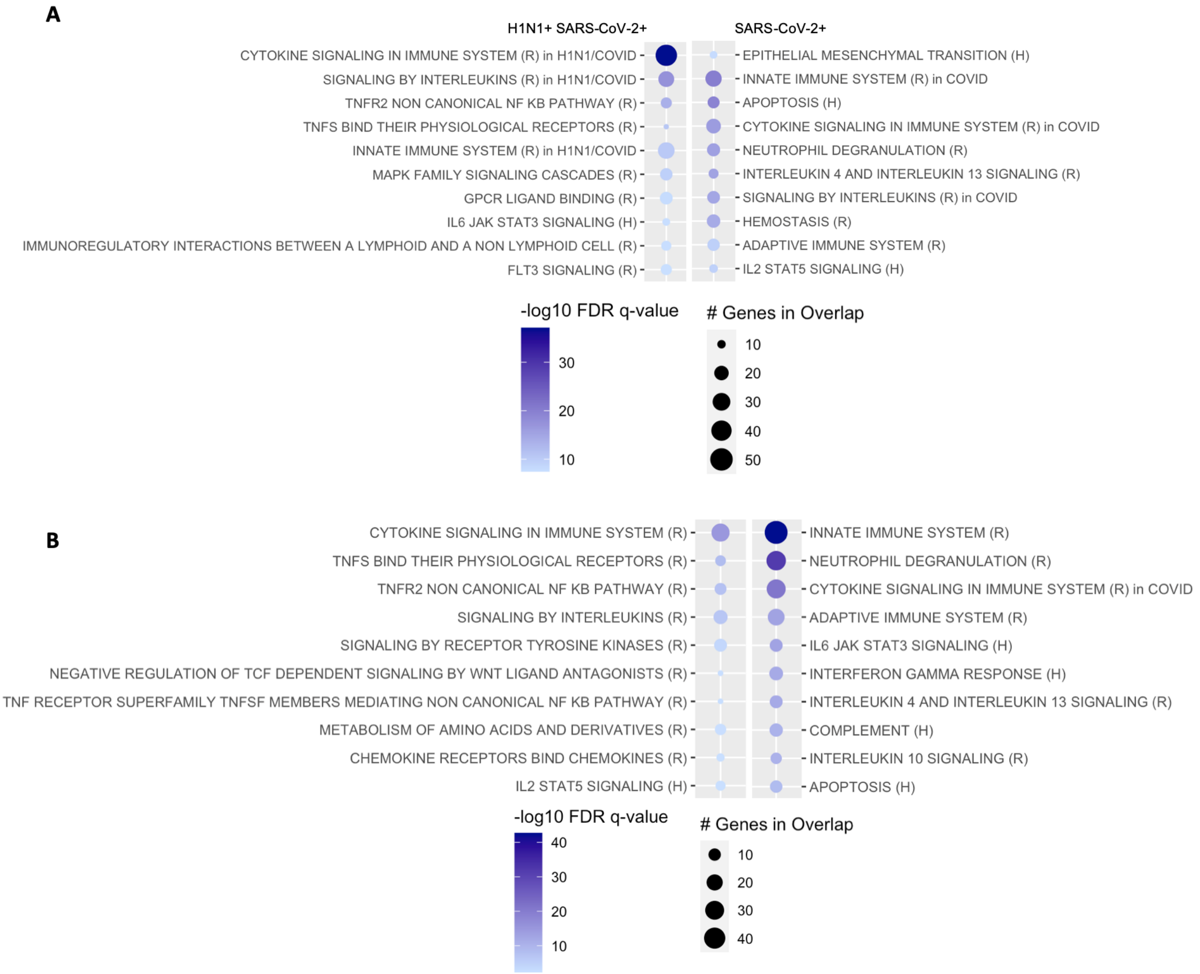

Fig. S5 related to Figures 3 and 4. SARS-CoV-2/H1N1 infected patient mimic H1N1 only gene expression. Geneset enrichment analysis using reactome (R) and hallmark (H) datasets for up-regulated or down-regulated genes in SARS-CoV-2 infected patients compared to SARS-CoV-2/H1N1 sample set for the epithelium **(A)** (technical replicates n= 12 for SARS-CoV-2 and n=5 SARS-CoV-2/H1N1), and macrophages **(B)** (technical replicates n= 9 for SARS-CoV-2 and n=3 SARS-CoV-2/H1N1). Differential gene expression was defined as p-value of 0.02 and log2 fold change of 0.5. SARS-CoV-2 infected patients (n=3) and SARS-CoV-2/H1N1 (n=1).

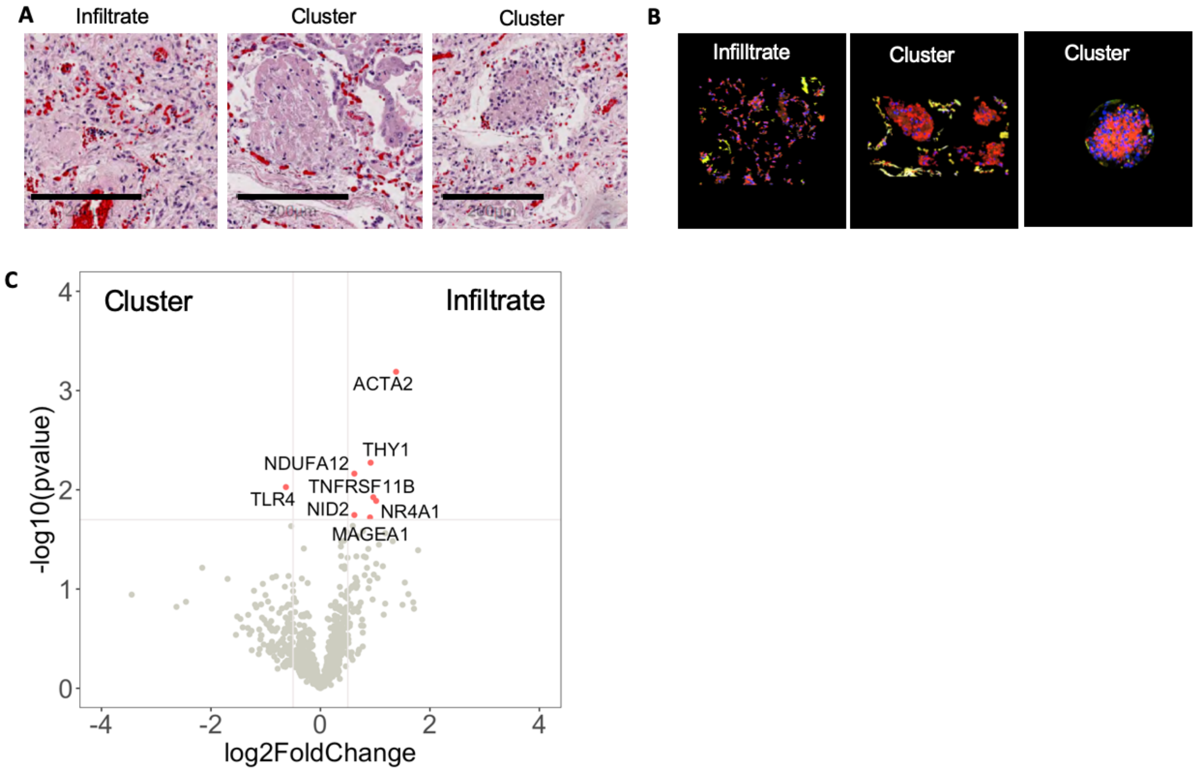

Fig. S6 related to Figure 4. Analysis of clustering macrophage transcriptional phenotype in SARS-CoV-2 patients. **(A)** Histological analysis of tissues sections stained by H&E revealed presence of clustering macrophages (scale bars = 200μm), which was confirmed by **(B)** CD68 staining (Blue = nuclei, Red = CD68, Yellow = EpCAM). Differential gene expression analysis of clustering vs infiltrating macrophages in SARS-CoV-2 infected patients showed only 8 differentially regulated genes. Differential gene expression was defined as p-value of 0.02 and log2 fold change of 0.5. SARS-CoV-2 infected patients (n=2). Technical replicates n= 4 for infiltrate and n=4 for cluster.

	Patient 1	Patient 2	Patient 3	Patient 4	Patient 5	Patient 6	Patient 7
**VIRAL TESTING**							
SARS-CoV-2	Positive	Positive	Positive	Negative	Negative	Negative	Positive
Influenza- H1N1	Negative	Negative	Negative	Positive	Positive	Positive	Positive
**DEMOGRAPHICS**							
Age in years	86	69	75	57	50	63	58
Race	C	AA	C	C	C	AA	AA
Gender	Male	Male	Male	Female	Female	Male	Female
**CO-MORBIDITIES**							
Diabetes Mellitus	None	Yes	None	None	None	None	None
CV Disease	None	None	None	None	HFpEF	None	None
Chronic Lung Disease	Asthma	None	None	None	COPD	None	CTD-ILD, COPD
Chronic Kidney Disease	None	None	None	None	None	None	None
**INTERVENTIONS**							
Antiviral (days)	None	None	Remdesivir (2)	Oseltamivir (7)	Oseltamivir (7)	Oseltamivir (10)	Oseltamivir (10)
Steroids (days)	MP (3)	HC (16)	Dex (1)	HC (3)	MP (2)	Pred (3)	HC (6)
Epoprostenol (days)	None	None	EPO 2d	None	EPO (16)	NO, EPO (3)	EPO (4)
Mechanical Ventilation	100% FiO2 via NRB	5.7cc/kg Tv, 12 PEEP, 60% FiO2	5.3cc/kg Tv, 16 PEEP, 60%FiO2	6cc/kg Tv, 16 PEEP, 100% FiO2	5.3cc/kg Tv, 18 PEEP, 100% FiO2	5.1cc/kg Tv, 15 PEEP, 90% FiO2	5.9cc/kg Tv, 10 PEEP, 100% FiO2
Proning (days)	None	Yes (9)	Yes (3)	None	Yes (5)	Yes (4)	None
ECMO (days)	None	None	None	VA (5)	None	None	None
**ICU ADMISSION**							
BMI (kg/m^2)	22.6	28	32.3	30	68.9	27.5	31
WBC (x10^3/cmm)	14.8	12.7	8.14	0.9	6.35	11.92	11.6
% PMN	81%	90%	88%	4%	87%	86%	84%
% Lymphocytes	6%	6%	7%	72%	7%	5%	9%
Troponin I (ng/L)	<0.3	88	17	38	128	n/a	1761
BNP (pg/mL)	n/a	78	536	445	581	n/a	299
AST (U/L)	34	106	68	15	41	32	43
Creatinine (mg/dL)	1.9	1.6	1.1	0.9	1.5	1.4	1.0
D-Dimer (ng/mL)	n/a	>20,000	921	2,978	n/a	n/a	12,301
PT (sec)	16.2	14.6	13.4	15.1	16.5	13.1	13.2
LDH (U/L)	n/a	777	n/a	198	n/a	n/a	472
**PHYSIOLOGY**							
Chest Radiograph (# of quadrants [1-4])	4	2	4	2	4	4	4
PaO2:FiO2 ratio	61	120	173	64	47	132	113
APACHE II Score (a) admission (b) 48h before death	(a) 20 (b) 24	(a) 26 (b) 35	(a) 20 (b) 43	(a) 17 (b) 24	(a) 13 (b) 37	(a) 20 (b) 38	(a) 14 (b) 17
SOFA Score (a) admission (b) 48h before death	(a) 5 (b) 6	(a) 12 (b) 18	(a) 10 (b) 17	(a) 10 (b) 17	(a) 11 (b) 14	(a) 7 (b) 15	(a) 7 (b) 7
**30-DAY**							
ICU Days	5	16	4	6	19	11	10
Ventilator Days	0	16	4	6	17	8	8
New Organ Failure	3	5	3	5	2	3	3

Table S1 related to STAR methods. Summary of patient demographics. Abbreviations: C: Caucasian; AA: African American; CV: cardiovascular; HFpEF: heart failure preserved ejection fraction; CTD-ILD: connective tissue disease associated interstitial lung disease; COPD: chronic obstructive lung disease; MP: Methylprednisone; HC: Hydrocortisone; TV: tidal volume, PEEP: positive end-expiratory pressure, FIO2: fraction of inspired oxygen, ECMO: extracorporeal membrane oxygenation, V-A: venous-arterial; BMI: body mass index; WBC: white blood cells; BNP: brain natriuretic

peptide; AST: aspartate aminotransferase; PT: prothrombin time; LDH: lactic acid dehydrogenase; PaO2: partial pressure arterial oxygen; APACHE: acute physiology and chronic health evaluation; SOFA: sequential organ failure assessment; ICU: intensive care unit.

COVID-19 added gene set

S	Spike Protein
ORF1ab	ORF1ab
ORF1ab_REV	Negative Strand ORF1ab
ACE2	angiotensin I converting enzyme 2
ABCA3	ATP binding cassette subfamily A member 3
AQP5	aquaporin 5
DHX58	DEXH (Asp-Glu-X-His) box polypeptide 58
FURIN	furin (paired basic amino acid cleaving enzyme)
HAS2	hyaluronan synthase 2
HOPX	HOP homeobox
IFNLR1	interferon lambda receptor 1
IL10RB	interleukin 10 receptor subunit beta
MUC13	mucin 13, cell surface associated
MUC2	mucin 2, oligomeric mucus/gel-forming
MUC5AC	mucin 5AC, oligomeric mucus/gel-forming
MUC5B	mucin 5B, oligomeric mucus/gel-forming
NAPSA	napsin A aspartic peptidase
PGC	progastricsin (pepsinogen C)
SCGB1A1	secretoglobin family 1A member 1
SFTPA1	surfactant protein A1
SFTPB	surfactant protein B
SFTPC	surfactant protein C
SFTPD	surfactant protein D
TP63	tumor protein p63
CC2D1B	coiled-coil and C2 domain containing 1B
SF3A3	splicing factor 3a subunit 3

Table S2 related to STAR methods. GeoMx spatial profiling SARS-CoV-2 gene set.
